# Cannabinoid Agonists Inhibit Neuropathic Pain Induced by Brachial Plexus Avulsion in Mice by Affecting Glial Cells and MAP Kinases

**DOI:** 10.1371/journal.pone.0024034

**Published:** 2011-09-13

**Authors:** Ana F. Paszcuk, Rafael C. Dutra, Kathryn A. B. S. da Silva, Nara L. M. Quintão, Maria M. Campos, João B. Calixto

**Affiliations:** 1 Department of Pharmacology, Centre of Biological Sciences, Universidade Federal de Santa Catarina, Florianópolis, Santa Catarina, Brazil; 2 Programa de Mestrado em Ciências Farmacêuticas, Universidade do Vale de Itajaí, Itajaí, Brazil; 3 School of Dentistry and Institute of Toxicology, Pontifícia Universidade Católica do Rio Grande do Sul, Porto Alegre, Brazil; Sapienza University of Rome, Italy

## Abstract

**Background:**

Many studies have shown the antinociceptive effects of cannabinoid (CB) agonists in different models of pain. Herein, we have investigated their relevance in neuropathic pain induced by brachial plexus avulsion (BPA) in mice.

**Methodology/Principal Findings:**

Mice underwent BPA or sham surgery. The mRNA levels and protein expression of CB_1_ and CB_2_ receptors were assessed by RT-PCR and immunohistochemistry, respectively. The activation of glial cells, MAP kinases and transcription factors were evaluated by immunohistochemistry. The antinociceptive properties induced by cannabinoid agonists were assessed on the 5^th^ and 30^th^ days after surgery. We observed a marked increase in CB_1_ and CB_2_ receptor mRNA and protein expression in the spinal cord and dorsal root ganglion, either at the 5^th^ or 30^th^ day after surgery. BPA also induced a marked activation of p38 and JNK MAP kinases (on the 30^th^ day), glial cells, such as microglia and astrocytes, and the transcription factors CREB and NF-κB (at the 5^th^ and 30^th^ days) in the spinal cord. Systemic treatment with cannabinoid agonists reduced mechanical allodynia on both the 5^th^ and 30^th^ days after surgery, but the greatest results were observed by using central routes of administration, especially at the 30^th^ day. Treatment with WIN 55,212-2 prevented the activation of both glial cells and MAP kinases, associated with an enhancement of CREB and NF-κB activation.

**Conclusions/Significance:**

Our results indicate a relevant role for cannabinoid agonists in BPA, reinforcing their potential therapeutic relevance for the management of chronic pain states.

## Introduction

Neuropathic pain, defined as “pain arising as a direct consequence of a lesion or disease affecting the somato-sensorial system” [1], is currently one of the most difficult types of pain to treat in the clinic. Persistent pain is often refractory to conventional analgesic therapy, with most patients obtaining, at best, only partial relief of symptoms [2]. In addition, most available pharmacological agents have their use limited by undesired effects or by drug interactions. Antidepressants and anticonvulsants have been demonstrated to provide analgesia, but they are effective in less than one half of patients [2]. Thus, the identification of novel therapeutic agents for the treatment of neuropathic pain is a crucial matter of interest.

There is considerable evidence supporting a role for cannabinoids in the modulation of pain, especially in neuropathic states [3], [4]. Endogenous cannabinoids and their receptors have been found to be expressed in key areas associated with pain processing, from peripheral sensory nerve endings to the spinal cord and in supraspinal centers [5], [6], and markedly increase in these areas in models of chronic pain [6]–[9]. The targets of cannabinoids are the two cloned receptor subtypes, denoted CB_1_ and CB_2_; both are members of the G protein-coupled receptor (GPCR) superfamily. CB_1_ receptor is mostly expressed in the central nervous system (CNS), particularly in the hippocampus, cortex, cerebellum, basal ganglia and spinal cord [5]. On the other hand, CB_2_ is primarily expressed in immune cells [10], but not exclusively outside the CNS [6], [9], [11].

Several studies have demonstrated the antinociceptive effects of cannabinoid receptor agonists in rat and mouse experimental models, including spontaneous, inflammatory and neuropathic pain [4], [6]. However, there is no evidence showing whether cannabinoids might modulate the neuropathic pain induced by brachial plexus avulsion (BPA). BPA usually occurs from high-speed motor vehicle accidents or birth palsy, and typically affects young men [12]. The management of BPA depends on the degree of damage and the site of injury, and requires a combination of surgical procedures and pharmacological approaches [12]. However, as in other neuropathic pain syndromes, the current therapy is unsatisfactory and produces critical collateral effects.

Our study evaluated the expression of cannabinoid receptors in the central nervous system, and the involvement of different signaling pathways implicated in nociception processing, such as glial cells, MAP kinases and transcription factors in the spinal cord structures of mice submitted to BPA. In addition, we analyzed the effects of either non-selective or selective cannabinoid receptor agonists in mechanical allodynia induced by BPA, and assessed whether cannabinoid agonists might modulate nociceptive signaling pathways to produce analgesia in this pain model.

## Results

### Neuropathic pain-like behavior induced by BPA

**Figure 1:**
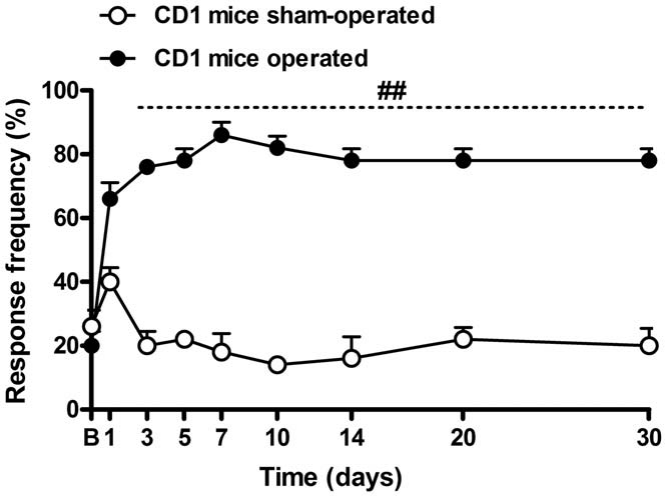
Mechanical allodynia induced by brachial plexus avulsion (BPA) in mice. Response of frequency of the right hindpaw assessed at several time-points by von Frey hair 0.4 g in sham-operated and operated (BPA) mice. Data are expressed as mean ± SEM (n = 4–6/group). ^##^p<0.01, significantly different from the sham-operated group (two-way ANOVA with Bonferroni's *post hoc* test). B, Baseline withdrawal threshold before surgery.

In this work, we observed the development of long-lasting mechanical allodynia in the right hindpaw of mice submitted to BPA observed until the 30^th^ day after surgery (two-way ANOVA, F = 11.24, df = 8 and p<0.001 for time), when compared to the sham-operated group, which did not develop differences in the mechanical threshold (Figure 1). However, the animals displayed good overall health after surgery, with standard locomotor activity throughout the experimental period of evaluation.

### Up-regulation of CB_1_ and CB_2_ receptor expression levels in the DRG and spinal cord after BPA

**Figure 2:**
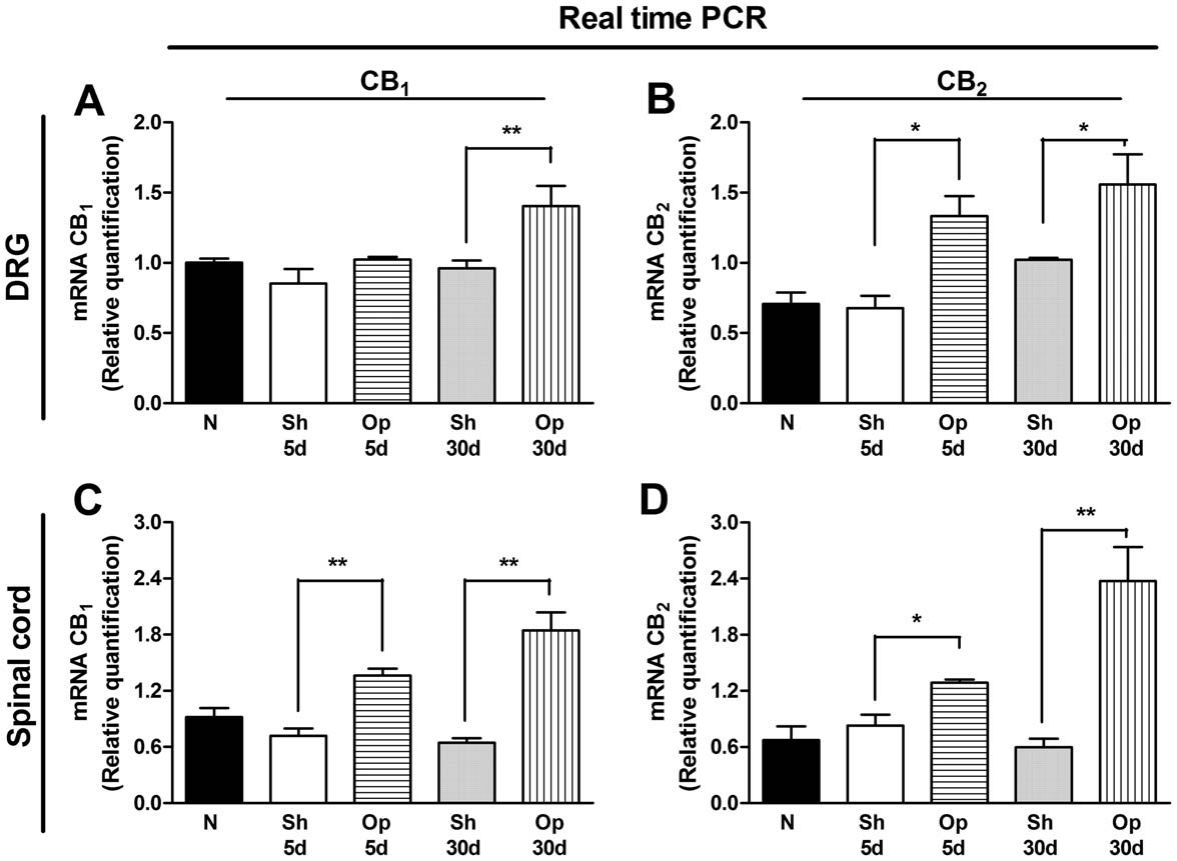
Evaluation of CB_1_ receptor (CB_1_) and CB_2_ receptor (CB_2_) mRNA levels in CNS structures of mice submitted to brachial plexus avulsion (BPA). The mRNA levels of the CB_1_ and CB_2_ receptors were evaluated in the dorsal root ganglia (DRG) (A and B) and dorsal horn of the spinal cord (C and D), respectively. GAPDH mRNA was used to normalize the relative amount of mRNA. N (naïve mice), Sh 5 d (sham-operated group 5 days after BPA), Op 5 d (operated group 5 days after BPA), Sh 30 d (sham-operated group 30 days after BPA) and Op 30 d (operated group 30 days after BPA). Data are expressed as mean ± SEM (n = 3/group), and are representative of three independent experiments. *p<0.05 and **p<0.01, significantly different from the respective sham-operated group (one-way ANOVA with Bonferroni's post hoc test).

**Figure 3:**
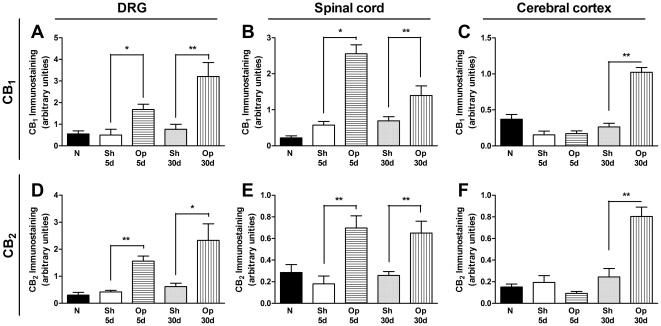
The CB_1_ receptor (CB_1_) and CB_2_ receptor (CB_2_) immunoreactivity in the dorsal root ganglion (DRG), the dorsal horn of the spinal cord and the cingulate cortex after brachial plexus avulsion (BPA) in mice. CB_1_ and CB_2_ receptor expression was evaluated in the DRG (A and D), the dorsal horn of the spinal cord (B and E) and the cingulate cortex (C and F) in N (naïve mice), Sh 5 d (sham-operated group 5 days after BPA), Op 5 d (operated group 5 days after BPA), Sh 30 d (sham-operated group 30 days after BPA) and Op 30 d (operated group 30 days after BPA). Immunostaining intensity and area were quantified by image analysis and are expressed as arbitrary units (mean ± SEM, n = 4/slices of 4–6/group).*p<0.05 and **p<0.01, significantly different from the respective sham-operated group (one-way ANOVA with Bonferroni's *post hoc* test).

As cannabinoid receptors play an important role in neuropathic pain [4], [6], we further evaluated the expression of both cannabinoid receptors after BPA in mice. In this set of experiments, we assessed the mRNA levels of CB_1_ and CB_2_ receptors in the DRG and spinal cord of mice submitted to BPA (at both the 5^th^ and 30^th^ days after the surgical procedure). The RT-PCR assay revealed constitutive expression of CB_1_ and CB_2_ receptors in both evaluated anatomical structures in naïve and sham-operated mice. Notably, a significant increase in CB_1_ receptor mRNA expression was observed only on the 30^th^ day after BPA in the DRG (one-way ANOVA, F = 7.44, df = 4, p<0.01), and on the 5^th^ and the 30^th^ days in the dorsal horn of the spinal cord of operated mice (one-way ANOVA, F = 20.24, df = 4, p<0.01) (Figure 2A and C). Of great interest, there was an increase in CB_2_ receptor mRNA levels in the DRG (one-way ANOVA, F = 6.63, df = 4, p<0.01) and in the dorsal horn of the spinal cord on the 5^th^ and 30^th^ (one-way ANOVA, F = 13.77, df = 4, p<0.05 and p<0.01) days after BPA (Figure 2B and D). These results were confirmed by immunohistochemical analysis of CB_1_ and CB_2_ receptor positivity in sections from the DRG and dorsal horn of the spinal cord of operated mice. Notably, our results also revealed a significant increase in the expression of both receptors CB_1_ and CB_2_ in regions of the DRG (one-way ANOVA, F = 12.57, df = 4, p<0.05 and F = 7.28, df = 4, p<0.01) (Figure 3A and D) and *dorsal* horn of the spinal cord associated mainly with the termination of nociceptive primary afferents (one-way ANOVA, F = 21.60, df = 4, p<0.05 and F = 6.18, df = 4, p<0.01) (Figure 3B and E), according to assessment on 5^th^ and 30^th^ days after surgery. In addition, there was an increase in the expression of both CB_1_ and CB_2_ receptors in the somatosensory cortex, especially in the cingulate cortex, of mice assessed 30 days after BPA (one-way ANOVA, F = 38.31, df = 4, p<0.05 and F = 24.38, df = 4, p<0.05) (Figure 3C and F, and Figure S1). Therefore, it is tempting to suggest that both CB_1_ and CB_2_ receptors are up-regulated after BPA in mice in several structures related to pain transmission and/or modulation, including the DRG, dorsal horn of the spinal cord and cingulate cortex.

### Increase in activation of glial cells, MAP kinases and the transcriptional factors CREB and NF-κB in the spinal cord after BPA

**Figure 4:**
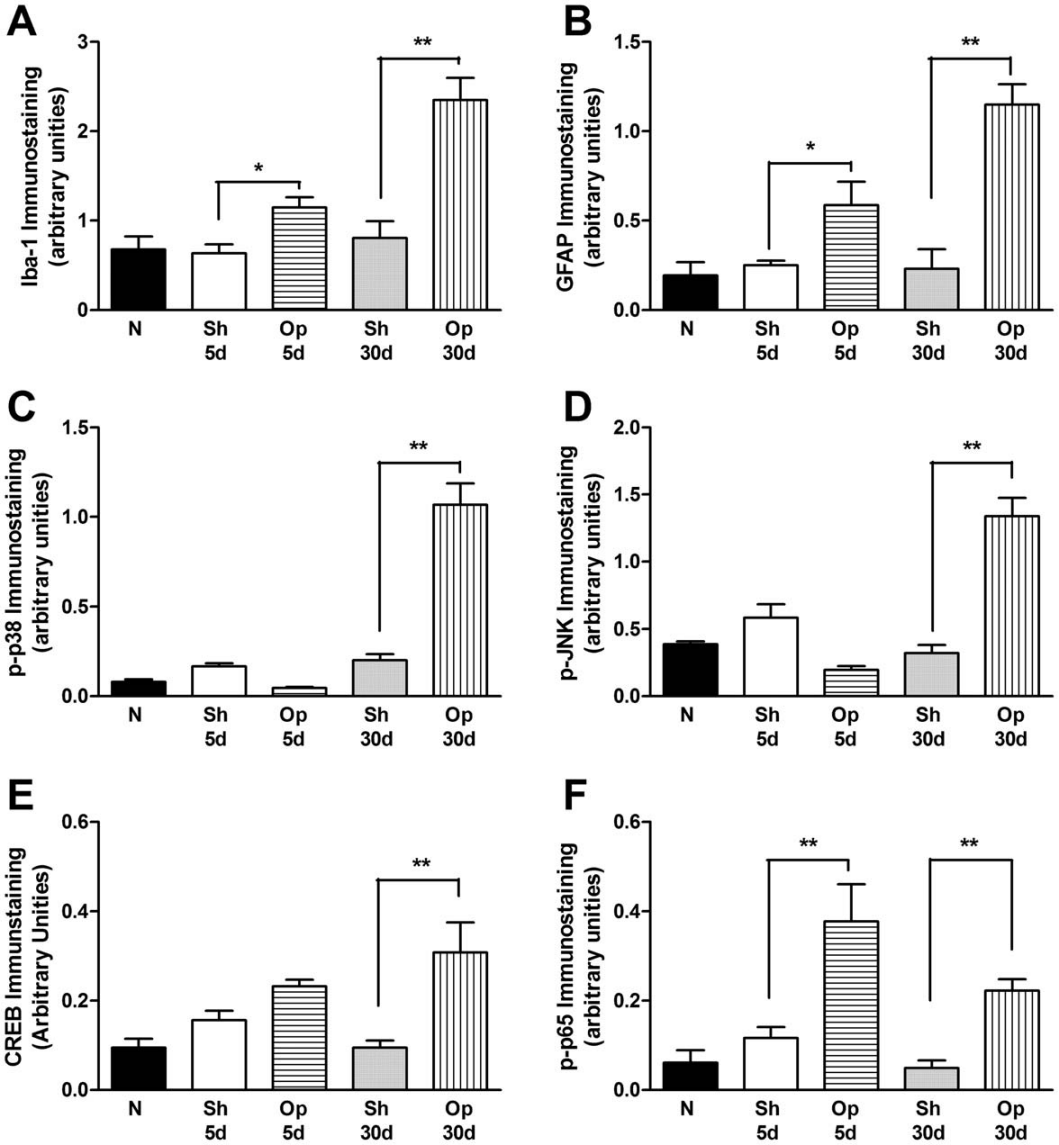
Brachial plexus avulsion (BPA)-induced mechanical hypersensitivity on the expression of glial cells, MAP kinases and transcription factors in the dorsal horn of the spinal cord in mice. Immunostaining of activated microglia (Iba-1, A), astrocytes (GFAP, B), phospho-p38 (p-p38, C) and phospho-JNK (p-JNK, D) MAP kinases, phospho-CREB (p-CREB, E) and phospho-p65 NF-κB (p-p65, F) transcription factors in N (naïve mice), Sh 5 d (sham-operated group 5 days after BPA), Op 5 d (operated group 5 days after BPA), Sh 30 d (sham-operated group 30 days after BPA) and Op 30 d (operated group 30 days after BPA). Immunostaining intensity and area were quantified by image analysis and are expressed as arbitrary units (mean ± SEM, n = 4/slices of 4–6/groups). *p<0.05 and **p<0.01, compared with sham-operated of the respective group and time (one-way ANOVA followed by Bonferroni's *post hoc* test). JNK: c-Jun N-terminal kinase; CREB: cAMP response element-binding protein; p65 NF-κB: nuclear phospho-p65 nuclear factor-κB (NF-κB).

Under normal conditions, glial cells are known for having a number of housekeeping functions in the central nervous system [13]. However, glial cells can contribute to neuropathic pain processing by the activation of intracellular pathways, such as the MAP kinase family and related transcription factors, which leads to an increase in inflammatory mediators and up-regulation of specific receptors [13]. According to these data, we further investigated some possible central mechanisms that could be involved in neuropathy induced by the BPA procedure. Figure 4 (A and B) shows that immunoreactivity for microglia (Iba-1) and astrocytes (GFAP) was increased on the 5^th^ and 30^th^ days after surgery (one-way ANOVA, F = 17.35, df = 4, p<0.01 and F = 15.08, df = 4, p<0.01) (Figure S2). Furthermore, phosphorylated p38 and JNK MAP kinases were markedly increased in the *dorsal* horn of the spinal cord, but only on the 30^th^ day after BPA (one-way ANOVA, F = 41.43, df = 4, p<0.01 and F = 24.77, df = 4, p<0.01), with no alterations in expression on the 5^th^ day (Figure 4C and D and Figure S2). Likewise, the transcription factor CREB was augmented on the 30^th^ day after BPA (one-way ANOVA, F = 6.83, df = 4, p<0.01) (Figure 4E and Figure S2), and the transcription factor NF-κB (p65) was increased on both the 5^th^ or 30^th^ (one-way ANOVA, F = 13.02, df = 4, p<0.05) days following surgery in the *dorsal* horn of the spinal cord (Figure 4F and Figure S2). The sham-operated mice did not display any significant alteration in immunoreactivity in comparison to the naïve group. Altogether, these data clearly demonstrate the importance of glial cells, protein kinases and different transcription factors in the modulation of BPA-induced neuropathic behavior.

### Systemic treatment with cannabinoid agonists inhibited neuropathic pain-like behavior induced by BPA

**Figure 5:**
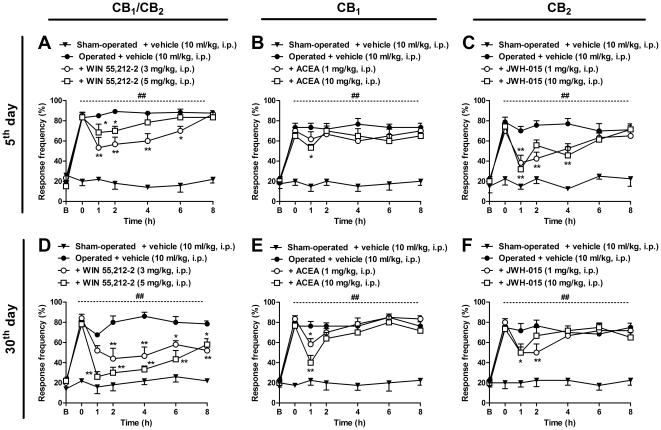
Systemic administration of cannabinoid agonists inhibits mechanical allodynia induced by brachial plexus avulsion (BPA) in mice. Response frequency of the right hindpaw assessed at several time-points in sham-operated and operated (BPA) mice treated with the non-selective CB_1_ and CB_2_ agonist, WIN 55,212-2 (3–5 mg/kg, i.p.), the selective CB_1_ agonist, ACEA (1–10 mg/kg, i.p.), the selective CB_2_ agonist, JWH-015 (1–10 mg/kg, i.p.) or vehicle (10 ml/kg, i.p.), administered on the 5^th^ (A, B and C) or 30^th^ (D, E and F) day after surgery. Data are expressed as mean ± SEM (n = 4–6/group). *p<0.05 and **p<0.01, significantly different from the operated group treated with vehicle (two-way ANOVA with Bonferroni's *post hoc* test). B, Baseline withdrawal threshold before surgery. 0, baseline withdrawal threshold after surgery on the day of the experiment (5^th^ and 30^th^ days).

An increasing body of evidence has emerged indicating that cannabinoids and their receptors are up-regulated in the CNS, and appear to be involved in neuropathic pain [6], [9]. Taking this into account, we next evaluated the effects of cannabinoid agonists in the mechanical allodynia induced by BPA. For this purpose, separate groups of animals were treated with the non-selective cannabinoid agonist WIN 55,212-2, the selective CB_1_ receptor agonist ACEA, or the selective CB_2_ receptor agonist JWH-015, all given by different routes of administration, on the 5^th^ or 30^th^ day after BPA. As shown in Figure 5 (A and D), mechanical allodynia was significantly reduced for 6 h after systemic administration of WIN 55,212-2 (3 mg/kg, i.p.), when given on the 5^th^ day post-surgery, with inhibition of 39±6%, based on the area under the curve (AUC) (two-way ANOVA, F = 62.32, df = 6 and p<0.001 for time, and F = 88.91, df = 3 and p<0.001 for treatment). In addition, WIN 55,212-2 administered on the 30^th^ day, either at doses of 3 or 5 mg/kg (i.p.) produced long-lasting inhibition (for 8 h after treatment) (two-way ANOVA, F = 46.36, df = 6 and p<0.01 for time, and F = 68.36, df = 3 and p<0.001 for treatment), with inhibition of 38±10% and 60±5%, respectively.

On the other hand, i.p. treatment with ACEA (10 mg/kg), a CB_1_ selective agonist, significantly reduced mechanical allodynia only when assessed 1 h after treatment, when administered on the 5^th^ day post-surgery (two-way ANOVA, F = 46.42, df = 6 and p<0.001 for time, and F = 37.39, df = 3 and p<0.001 for treatment); nonetheless, this did not produce a significant inhibition based on the AUC (Figure 5B). Conversely, on the 30^th^ day, both doses of ACEA significantly reduced mechanical allodynia 1 h after treatment (two-way ANOVA, F = 53.33, df = 6 and p<0.001 for time, and F = 73.92, df = 3 and p<0.001 for treatment), but with a slight effect according to the AUC (Figure 5E). In addition, on the 5^th^ day after BPA, the selective CB_2_ receptor agonist JWH-015 (1 and 10 mg/kg), given by the i.p. route, was able to significantly reduce mechanical allodynia (two-way ANOVA, F = 28.83, df = 6 and p<0.001 for time, and F = 44.12, df = 3 and p<0.001 for treatment) (Figure 5C). On the other hand, JWH-015 (1 and 10 mg/kg, i.p.) slightly reduced BPA-induced allodynia within the first two hours of administration, and did not produce any significant effect when dosed 30 days after surgery (two-way ANOVA, F = 28.41, df = 6 and p<0.001 for time, and F = 75.91, df = 3 and p<0.001 for treatment, Figure 5F). It is important to mention that under these treatment protocols, none of the cannabinoid treatments produced any significant motor deficit, no typical signs of catalepsy or hypothermia, and failed to change the basal threshold of pain, according to the tetrad test used to evaluate cannabinoid-related central effects (Supplementary Methods and Table S1).

### Local treatment with cannabinoid agonists failed to inhibit neuropathic pain-like behavior induced by BPA

In order to assess the involvement of local activation of the cannabinoid system following BPA-induced mechanical hypersensitivity, separate groups of mice received peripheral injection of the non-selective (WIN 55,212-2), the CB_1_ selective (ACEA) or the CB_2_ selective (JWH-015) cannabinoid agonists by the i.pl. route. However, the agonists WIN 55,212-2 (10 and 30 µg/paw) and JWH-015 (10 and 30 µg/paw), administered either on the 5^th^ or 30^th^ day after surgery, did not produce significant changes in mechanical allodynia induced by BPA in mice (Figure S3). On the other hand, when the CB_1_ selective agonist ACEA (30 µg/paw) was administered on the 5^th^ day after surgery, it significantly reduced mechanical allodynia until 2 h after treatment (two-way ANOVA, F = 57.96, df = d and p<0.001 for time, and F = 74.88, df = 3 and p<0.001 for treatment); however, this did not result in significant inhibition when calculated based on the AUC. Contrarily, on the 30^th^ day after surgery, ACEA treatment did not reduce mechanical allodynia induced by BPA.

### Neuropathic pain-like behavior induced by BPA was greatly inhibited by central treatment with cannabinoid agonists

**Figure 6:**
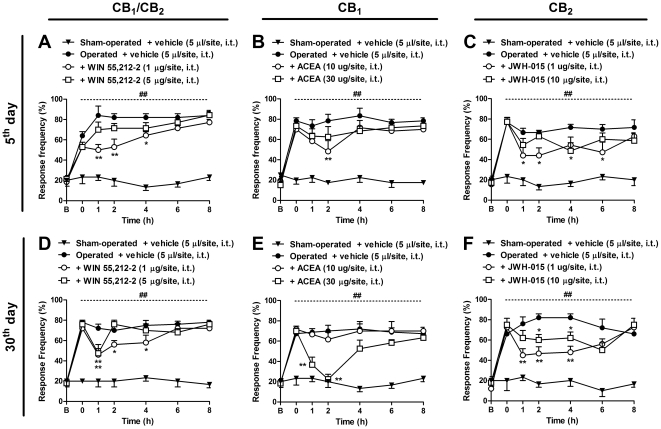
Effects of spinal (i.t.) administration of cannabinoid agonists on mechanical allodynia induced by brachial plexus avulsion (BPA) in mice. Response frequency of the right hindpaw assessed at several time-points in operated (BPA) mice treated with the non-selective CB_1_ and CB_2_ agonist, WIN 55,212-2 (1–5 µg/site, i.t.), the selective CB_1_ agonist ACEA (10–30 µg/site, i.t.), the selective CB_2_ agonist, JWH-015 (1–10 µg/site, i.t.) or vehicle (5 µl/site, i.t.), administered on the 5^th^ (A, B and C) or 30^th^ (D, E and F) day after surgery. Data are expressed as mean ± SEM (n = 4–6/group). *p<0.05 and **p<0.01, significantly different from the operated group treated with vehicle (two-way ANOVA with Bonferroni's *post hoc* test). B, Baseline withdrawal threshold before surgery. 0, baseline withdrawal threshold after surgery on the day of the experiment (5^th^ and 30^th^ days).

Next, to verify the possible involvement of spinal pathways in the modulating actions of cannabinoids following BPA, animals were treated with the cannabinoid agonists by the i.t route. As depicted in Figure 6A and C, i.t. treatment with WIN 55,212-2 (1 µg/site) and JWH-015 (1 µg/site) significantly reduced mechanical allodynia induced by BPA for 4 h (two-way ANOVA, F = 45.33, df = 6 and p<0.001 for time, and F = 37.57, df = 3 and p<0.001 for treatment) or 6 h (two-way ANOVA, F = 22.11, df = 6 and p<0.001 for time, and F = 19,84, df = 3 and p<0.001 for treatment) after treatment, respectively. On the other hand, i.t. treatment with ACEA (10 µg/site), given 5 days after surgery, showed a slight reduction in mechanical allodynia caused by BPA (Figure 6B) (two-way ANOVA, F = 43.95, df = 6 and p<0.001 for time, and F = 31.82, df = 3 and p<0.001 for treatment). When evaluated on the 30^th^ day after BPA, the administration of WIN 55,212-2 (1 µg/site) significantly reduced mechanical allodynia until 4 h after treatment (two-way ANOVA, F = 52.35, df = 6 and p<0.001 for time, and F = 44.05, df = 3 and p<0.001 for treatment) (Figure 6D). Likewise, treatment with ACEA (30 µg/site) almost abolished mechanical allodynia for 2 h after treatment (Figure 6E) (two-way ANOVA, F = 46.91, df = 6 and p<0.001 for time, and F = 16.64, df = 2 and p<0.001 for treatment). Similarly, the treatment with JWH-015 (1 and 10 µg/site) on the 30^th^ day after BPA produced a marked and significant reduction in mechanical nociceptive responses for 4 h after treatment (two-way ANOVA, F = 24.53, df = 6 and p<0.001 for time, and F = 79.83, df = 3 and p<0.001 for treatment), with inhibition of 44±9% and 30±7%, respectively (Figure 6F).

**Figure 7:**
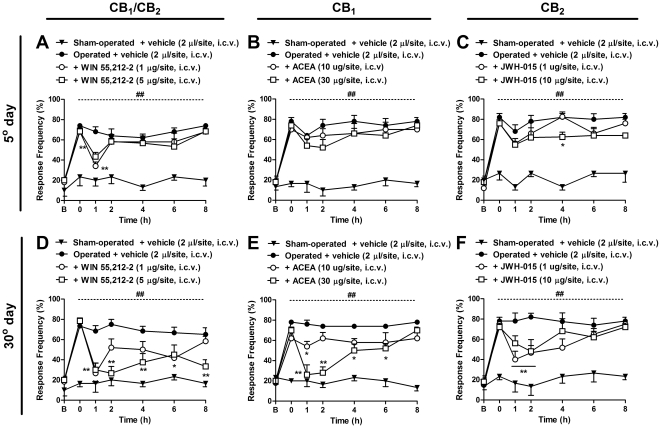
Effects of supraspinal (i.c.v.) treatment with cannabinoid agonists on mechanical allodynia induced by brachial plexus avulsion (BPA). Response frequency of the right hindpaw assessed at several time-points in operated (BPA) mice treated with the non-selective CB_1_ and CB_2_ agonist, WIN 55,212-2 (1–5 µg/site, i.c.v.), the selective CB_1_ agonist, ACEA (10–30 µg/site, i.c.v.), the selective CB_2_ agonist, JWH-015 (1–10 µg/site, i.c.v.) or vehicle (2 µl/site, i.c.v.), administered on the 5^th^ (A, B and C) or 30^th^ (D, E and F) day after surgery. Data are expressed as mean ± SEM (n = 4–6/group). *p<0.05 and **p<0.01, significantly different from the operated group treated with vehicle (two-way ANOVA with Bonferroni's *post hoc* test). B, Baseline withdrawal threshold before surgery. 0, baseline withdrawal threshold after surgery on the day of the experiment (5^th^ and 30^th^ days).

In another set of experiments, the cannabinoid agonists were administered by the i.c.v. route to verify the possible supraspinal role of cannabinoids in the neuropathic pain-like behavior associated with BPA. As shown in Figure 7 (A and C), i.c.v. treatment with WIN 55,212-2 (1 and 5 µg/site) and JWH-015 (10 µg/site) on the 5^th^ day following surgery slightly, but significantly, reduced allodynia induced by BPA for 1 h (two-way ANOVA, F = 37.28, df = 6 and p<0.001 for time, and F = 27.37, df = 3 and p<0.001 for treatment) and 4 h (two-way ANOVA, F = 40.83, df = 6 and p<0.001 for time, and F = 38.29, df = 3 and p<0.001 for treatment) after treatment, respectively. Surprisingly, treatment with ACEA (10 and 30 µg/site) did not reduce the nociceptive response when administered on the 5^th^ day after surgery. However, i.c.v. administration of WIN 55,212-2 (Figure 7D), ACEA (Figure 7E) and JWH-015 (Figure 7F) 30 days after the surgical procedure markedly reduced mechanical allodynia induced by BPA for up to 8 h (two-way ANOVA, F = 23.84, df = 6 and p<0.001 for time, and F = 30.28, df = 3 and p<0.001 for treatment), 6 h (two-way ANOVA, F = 24.99, df = 6 and p<0.001 for time, and F = 50.67, df = 3 and p<0.001 for treatment) and 2 h (two-way ANOVA, F = 25.09, df = 6 and p<0.001 for time, and F = 20.45, df = 3 and p<0.001 for treatment) after treatment with the highest dose, respectively. This collection of data clearly demonstrates that both CB_1_ and CB_2_ receptors might be distinctly up-regulated in different areas of the CNS in a time-dependent manner.

Taken together, this series of experimental evidence suggests that on the 5^th^ day, either peripheral or spinal cannabinoid receptors seem to be related to the onset and maintenance of mechanical allodynia induced by BPA. On the other hand, on the 30^th^ day, i.t. and i.c.v. treatment with the cannabinoid agonists showed greater inhibition of mechanical allodynia induced by BPA which suggests, at least in part, the principal action of spinal and supra-spinal cannabinoid receptors in the persistence of allodynia induced by BPA.

### Effect of cannabinoid antagonists in the analgesic response induced by WIN 55,212-2 in mice

**Figure 8:**
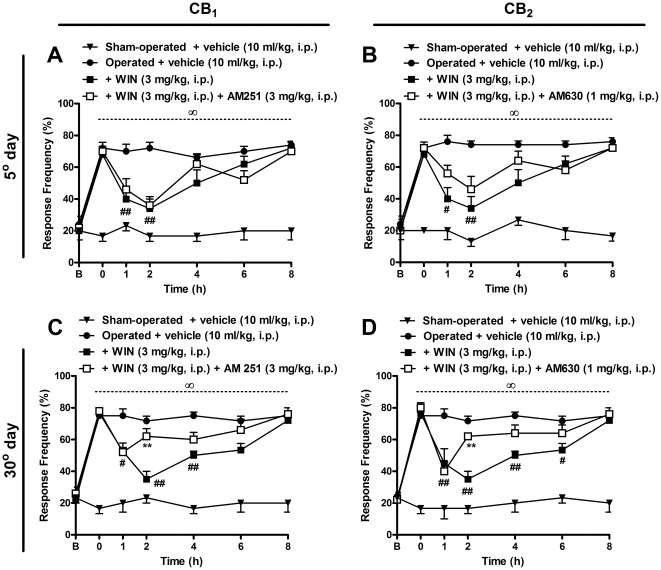
Effects of pre-treatment with selective cannabinoid antagonists, administered on the 5^th^ and 30^th^ days after brachial plexus avulsion (BPA), on analgesia produced by WIN 55,212-2 treatment in mice. Response frequency of the right hindpaw assessed at several time-points in operated (BPA) mice treated with vehicle (10 ml/kg, i.p.) or a CB_1_-selective antagonist (AM 251, 3 mg/kg, i.p.) (A and C) or a CB_2_-selective antagonist (AM 630, 1 mg/kg, i.p.) (B and D) 30 min before systemic treatment with the non-selective agonist, WIN 55,212-2 (3 mg/kg, i.p.) administered on the 5^th^ (A and B) or 30^th^ (C and D) day after surgery. Data are expressed as mean ± SEM (n = 4–6/group). ^#^p<0.01, significantly different from the operate-vehicle-treated group. **p<0.01, significantly different from the WIN 55,212-2 treated group, and ^∞^p<0,01 is significantly different from sham-operated treated with vehicle (two-way ANOVA with Bonferroni's *post hoc* test).

To confirm if the analgesic effects of cannabinoid agonists are due to specific receptor activation, we included a group of experiments using the selective CB_1_ (AM 251, 3 mg/kg, i.p.) or CB_2_ (AM 630, 1 mg/kg, i.p.) antagonists before WIN 55,212-2 (3 mg/kg, i.p.) administration. The results depicted in Figure 8B demonstrate a minor reversion of WIN 55,212-2-induced analgesia in the CB_2_-selective antagonist-treated group on the 5^th^ day after BPA (two-way ANOVA, F = 27.82, df = 6 and p<0.001 for time, and F = 43.86, df = 3 and p<0.001 for treatment). On the other hand, the treatment with the CB_1_-selective antagonist did not prevent the reduction of mechanical allodynia produced by WIN 55,212-2 treatment at this experimental time-point (Figure 8A). Moreover, when different groups of mice were treated with the CB_1_-selective antagonists (two-way ANOVA, F = 43.53, df = 6 and p<0.001 for time, and F = 87.32, df = 3 and p<0.001 for treatment) or CB_2_-selective antagonists (two-way ANOVA, F = 34.96, df = 6 and p<0.001 for time, and F = 182.3, df = 3 and p<0.001 for treatment) on the 30^th^ day after surgery, both drugs were able to markedly reverse the inhibition produced by WIN 55,212-2 (3 mg/kg, i.p.) treatment for 2 h after antagonist treatment (Figure 8C e D).

### Effect of cannabinoid agonist treatment on knockdown of CB_1_ and CB_2_ in mice

**Figure 9:**
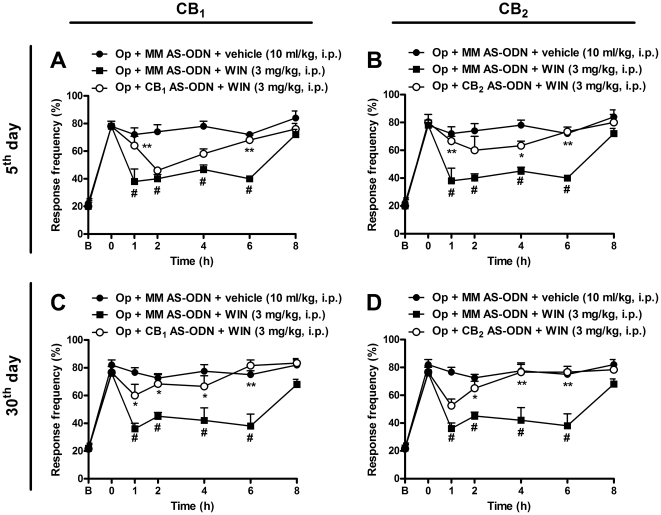
Effect of treatment with antisense oligodeoxinucleotide (AS-ODN) against the CB_1_ or CB_2_ receptor on mechanical allodynia induced by brachial plexus avulsion (BPA) in mice. Different groups of operated (Op) mice received an i.t. injection twice daily of mismatch AS-ODN (12.5 µg/site), CB_1_ AS-ODN (12.5 µg/site) or CB_2_ AS-ODN (12.5 µg/site). Systemic administration of WIN 55,212-2 (3 mg/kg, i.p.) produced a significant reduction of mechanical allodynia induced by BPA on the 5^th^ (A and B) and 30^th^ (C and D) days after surgery. The antinociceptive activity of WIN 55,212-2 (3 mg/kg, i.p.) was inhibited by both CB_1_ AS-ODN (12.5 µg/site, i.t.) and CB_2_ AS-ODN (12.5 µg/site, i.t.) on the 5^th^ (A and C) and 30^th^ (B and D) days after surgery, respectively. AS-ODN were administered on three consecutive days starting on the 2^nd^ and 27^th^ days after BPA surgery. The mice which received mismatch (MM) AS-ODN demonstrated similar responses throughout the testing period. Data are expressed as mean ± SEM (n = 4–6/group). *p<0.05 and **p<0.01, significantly different from the operated group that received MM AS-ODN and treated with WIN 55,212-2 (two-way ANOVA with Bonferroni's *post hoc* test). ^#^p<0.01, significantly different from operated-mice group (Op) that received MM AS-ODN and treated with vehicle. B, Baseline withdrawal threshold before surgery. 0, baseline withdrawal threshold after surgery on the day of the experiment (5^th^ and 30^th^ days).

Next, to verify the possible involvement of spinal cannabinoid receptors in the modulation of mechanical allodynia induced by BPA, we performed intrathecal pre-treatment with selective CB_1_ and CB_2_ antisense oligodeoxynucleotides (AS-ODN) used to knock down the expression of these receptors in the lumbar spinal cord after BPA. The group of mice that received the mismatch control ODN (MM AS-ODN) did not demonstrate interference in the pain threshold, and the treatment with the cannabinoid agonist WIN 55,212-2 (3 mg/kg, i.p.) produced a significant reduction in mechanical allodynia induced by BPA (two-way ANOVA, F = 41.49 df = 6 and p<0.001 for time, and F = 52.50, df = 1 and p<0.001 for treatment) when assessed on the 5^th^ or 30^th^ day after surgery (Figure 9A–D). Figure 9A shows that treatment of mice with the CB_1_ AS-ODN (12.5 µg/site, 2× day, i.t.) delayed by 1 h the inhibition produced by WIN 55,212-2 (3 mg/kg, i.p.) pre-treatment (two-way ANOVA, F = 65.25, df = 6 and p<0.001 for time, and F = 26.40, df = 2 and p<0.001 for treatment), when administered for three consecutive days and evaluated on the 5^th^ day after BPA. However, when the animals were treated for three consecutive days and evaluated on the 30^th^ day after BPA, the CB_1_ AS-ODN almost completely prevented (for 6 h after treatment) the analgesia produced by WIN 55,212-2 (3 mg/kg, i.p.), with an inhibition of 90±20% (two-way ANOVA, F = 49.08, df = 6 and p<0.001 for time, and F = 16.35, df = 2 and p<0.001 for treatment) (Figure 9C). A similar result was observed when mice received the CB_2_ AS-ODN (12.5 µg/site, 2× day, i.t.) for three consecutive days and were evaluated on the 5^th^ and 30^th^ days after BPA, showing an inhibition in WIN 55,212-2-induced analgesia for 6 h after treatment, with inhibition of 70±7% (two-way ANOVA, F = 50.77, df = 6 and p<0.001 for time, and F = 30.01, df = 2 and p<0.001 for treatment) and 91±15% (two-way ANOVA, F = 56.48, df = 6 and p<0.001 for time, and F = 29.68, df = 2 and p<0.001 for treatment), respectively (Figure 9B and D).

Extending the functional evidence, treatment with the AS-ODN was able to diminish the expression of both CB_1_ and CB_2_ receptors in the mouse spinal cord, when compared with the group treated with MM AS-ODN, evaluated by immunohistochemical analysis (Figure S4). These results suggest a major participation of the CB_2_ receptor on the 5^th^ day after surgery, when a large inflammatory response is involved and crosstalk of both CB_1_ and CB_2_ receptors on the 30^th^ day after surgery, when central sensitization is involved.

### Effects of cannabinoid agonists on the activation of glial cells, MAP kinases, and transcription factors in the spinal cord after BPA

**Figure 10:**
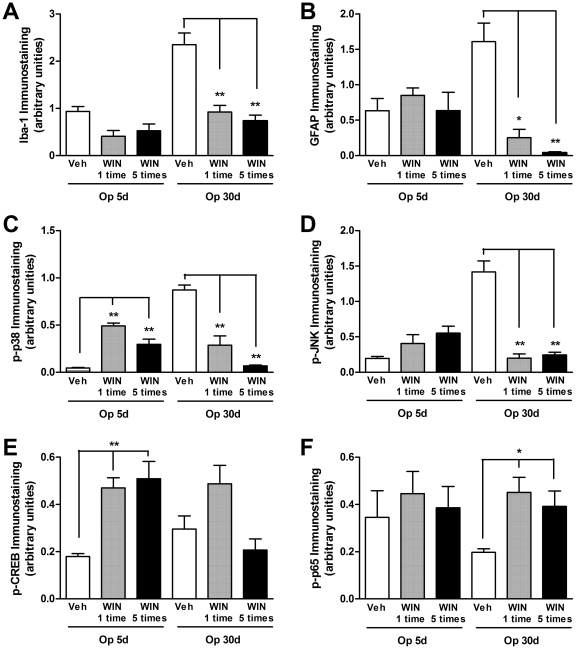
Effect of cannabinoid agonist on the expression of glial cells, MAP kinases and transcription factors in the dorsal horn of the spinal cord after brachial plexus avulsion (BPA) in mice. Effects of acute (once) or long-term (five times, 12×12 h) treatment with WIN 55,212-2 (3 mg/kg, i.p.) on the 5^th^ or 30^th^ days after BPA on the expression of activated microglia (Iba-1, A), astrocytes (GFAP, B), phospho-p38 (p-p38, C), phospho-JNK (p-JNK, D), phospho-CREB (p-CREB, E) and phospho-p65 NF-κB (p-p65, F). Immunostaining intensity and area were quantified by image analysis and are expressed as arbitrary units (mean ± SEM, n = 4/slices of 4–6/groups). *p<0.05 and **p<0.01, compared with sham-operated of respective group and time (one-way ANOVA followed by Bonferroni's *post hoc* test). JNK: c-Jun N-terminal kinase; CREB: cAMP response element-binding protein; p65 NF-κB: nuclear phospho-p65 nuclear factor-κB (NF-κB).

In order to further evaluate the possible mechanisms involved in the analgesic actions of cannabinoid agonists, we next assessed whether treatment with WIN 55,212-2 was able to decrease the activation and/or expression of glial cells, as well as the activation MAP kinases and transcription factors in the spinal cord of mice on the 5^th^ or 30^th^ day after BPA. Of high interest, acute (single administration) or prolonged treatment (five administrations in a 12/12 h schedule of treatment) with the non-selective cannabinoid agonist WIN 55,212-2 (3 mg/kg, i.p.) markedly reduced the increased activation of microglia (60±9% and 68±5%, respectively) (one-way ANOVA, F = 19.96, df = 5, p<0.001, Figure 10A), astrocytes (84±7% and 97±1%, respectively) (one-way ANOVA, F = 7.16, df = 5, p<0.001, Figure 10B), phospho-p38 (67±11% and 92±2%, respectively) (one-way ANOVA, F = 19.14, df = 5, p<0.001, Figure 10C) and phospho-JNK (86±4% and 82±3%, respectively) (one-way ANOVA, F = 24.04, df = 5, p<0,001, Figure 10D), only on the 30^th^ day (Figure S5) after surgery. On the other hand, there were an increase in the activation of phospho-p38 and CREB following the pre-treatment of animals with WIN 55,212-2 (3 mg/kg, i.p.) on the 5^th^ day after surgery (Figure 10E) and an increase in the p65 subunit of NF-κB 30 days after BPA (Figure 10F and Figure S5) (ANOVA, *p*<0.05). Taken together, this series of experimental evidence suggests that the analgesic effects of cannabinoid agonists on neuropathic hypersensitivity depend, at least in part, on the ability of these compounds to inhibit glial cells, mainly microglia and astrocytes via the down-regulation of MAP kinase activation.

## Discussion

In this study, we demonstrate for the first time that mechanical allodynia induced by BPA in mice is regulated by either systemic or central treatment with both CB_1_ and CB_2_ cannabinoid agonists. The pharmacological data correlate well with a marked up-regulation of both CB_1_ and CB_2_ receptors in the DRG, the spinal cord and the somatosensory cortex (cingulate cortex) after BPA. Our data also show that the mechanisms responsible for cannabinoid agonist-mediated analgesic actions are primarily associated with the ability of these compounds to prevent the activation of microglia and astrocytes cells, and to inhibit the phosphorylation of MAP kinases, such as p38 and JNK, associated with an increase in the expression of the transcription factors CREB and NF-κB. Additionally, our results also support a major involvement of CB_2_ receptors in the acute phase (5^th^ day) and both CB_1_ and CB_2_ receptors in the chronic phase (30^th^ day) of neuropathic pain induced by BPA.

In models of chronic pain, several studies have suggested the up-regulation of both cannabinoid receptors in different areas of the central nervous system [6]–[9]. The results of the present study demonstrate that there is a basal mRNA expression level of both CB_1_ and CB_2_ receptors in the DRG and spinal cord of naïve mice, and also demonstrate up-regulation of both receptors on the 5^th^ and 30^th^ days after BPA surgery in mice. However, we are not currently able to define in what cell type the up-regulation of cannabinoid receptors takes place, and further studies are necessary to clarify this point. Previous studies have demonstrated an increase in CB_2_ receptor expression in non-neuronal cells [10] and in central neurons after nerve injury [6], [11]. In the same way, the expression of CB_1_ receptors has been observed in neuronal cells and in a smaller proportion of non-neuronal cells, chiefly after nerve injury [5], [11]. Moreover, earlier studies have demonstrated that in models of chronic pain, there is an increase in the production of endocannabinoids in central areas, especially in the spinal cord [11]. Therefore, we might suggest that increased expression of cannabinoid receptors is one of the pathways activated to control BPA-related neuropathic pain.

The increase in cannabinoid receptor expression observed after BPA surgery was aligned with the augmented activation of microglia and astrocytes in the spinal cord of mice, on both the 5^th^ and 30^th^ days after BPA. It has been reported that activation of glial cells in the central nervous system contributes to aberrant pain behavior and plays an important role in central sensitization of central neurons [13]. Although we are not able to demonstrate the localization of cannabinoid receptor expression in the present study, many studies have demonstrated that increased CB_2_ receptor expression after nerve injury is chiefly related to microglial activation [10], while CB_1_ receptors have been found in many types of cells, including microglia, astrocytes and neurons in the spinal cord of rodents [5], [11], [14]. The involvement of glia in neuropathic pain has been confirmed by treatment with minocycline, an inhibitor of activated microglia, which significantly reduces mechanical and thermal hyperalgesia in different models of neuropathy in rodents [15]. In this work, we demonstrated that acute and prolonged treatment with WIN 55,212-2 reduced the activation of both microglia and astrocytes on the 30^th^ day after surgery, suggesting that cannabinoid agonists are involved in glial cell regulation.

Recent research on central sensitization following nerve injury has indicated a relevant role for the MAP kinases JNK and p38 in nociceptive hyper-reactivity [16], [17]. In fact, JNK and p38 activation was found to be increased in animal models of acute and persistent pain [16], [17]. Some studies have suggested that inhibition of p38 prevents mechanical allodynia and the development of neuropathic pain in rodents [16], and that JNK inhibitors can reverse mechanical allodynia induced in neuropathic models [17]. The mechanical allodynia observed in the BPA model was also preceded by a marked activation of both MAP kinases in the spinal cord of mice, according to the assessment performed 30 days after surgery. Additionally, both acute and long-term treatment with WIN 55,212-2 reduced the activation of JNK and p38 MAPKs when observed on the 30^th^ day after surgery. These results suggest that MAP kinases such as JNK and p38 play a pivotal role in the control of neuronal hyperexcitability and neuropathic pain after BPA, when central sensitization is involved. However, on the 5^th^ day after surgery, an increase in p38 phosphorylation occurred after acute and long-term treatment with WIN 55,212-2. It is known that both cannabinoid receptors are able to phosphorylate and activate different MAPK members, inducing the transcription of genes with different effects on cell survival [18]. So, the increase in p38 activation could be related to the protective role of MAPK against the processes of neuropathic pain induction in the spinal cord. However, further studies are necessary to investigate this hypothesis.

In neuropathic pain, numerous pro-inflammatory cytokines or neurotransmitters are produced and released in the spinal cord after injury, and in turn lead to increased CREB and NF-κB phosphorylation in superficial dorsal horn neurons, contributing to neuropathic pain [19], [20]. In the BPA model, there was a great increase in CREB phosphorylation in the spinal cord on the 30^th^ day after surgery. In addition, an increase in NF-κB activation was observed in the spinal cord of mice on both the 5^th^ and 30^th^ days after BPA. This set of results suggests that the BPA model produces alterations in the spinal cord that involve glial cell activation and an increase in MAP kinases activation through the activation of transcription factors, such as CREB and NF-κB, that could contribute to the genesis and maintenance of pain after nerve injury. Intriguingly, the activation of the transcription factors CREB and p65 NF-κB observed in the spinal cord after BPA was significantly increased by acute or prolonged treatment with WIN 55,212-2 on the 5^th^ and the 30^th^ days after BPA, respectively. Despite their participation in some pathological states like neuropathic pain, both transcription factors regulate additional cellular responses, including proliferation, survival and differentiation [20], [21]. So, the increase in CREB and p65 NF-κB activation by WIN 55,212-2 treatment could induce a protective effect that needs to be investigated further. There are published data showing that cannabinoid agonists are able to increase CREB phosphorylation, an effect that seems to be important for regulating the growth of neurites in cultured neuronal cells [22], [23]. In addition, another study has demonstrated that NF-κB has a dual role in the CNS: its activation in neurons is involved in neuronal survival and neuritic growth depending on the degree and site of activation, whereas activation in glial cells promotes the release of inflammatory mediators, and thereby contributes to the inflammatory process [24]. Taken together, the activation of CREB and p65 NF-κB may have a beneficial effect in terms of the cannabinoid effect on neuropathic pain induced by BPA.

Several studies have demonstrated that cannabinoids might act as analgesics [3], [4], and it has been demonstrated that the antinociceptive effects of cannabinoids are predominantly mediated through CB_1_ receptors [8], [25]. However, many studies have pointed out CB_2_ receptor agonists as new therapeutic options to treat chronic pain without the psychotropic effects produced by CB_1_ receptor agonists [9], [26]. Our results revealed the participation of CB_2_ receptors in the initial phase of neuropathic pain associated with BPA, and suggest crosstalk between CB_1_ and CB_2_ receptors in the late phase of neuropathy. The participation of CB_2_ receptors in initial phase of neuropathic pain in the BPA model could be related to its known anti-inflammatory activity, probably acting in immune cells in the periphery and in glial cells in the spinal cord. Recent studies have reported that CB_2_ receptor stimulation reduces microglial activation and the release of inflammatory mediators such as cytokines and chemokines [9], [27]. However, further studies are required to confirm the cell type related to the CB_2_ receptor-mediated effects in the BPA model.

Our data also suggest that CB_1_ receptors can participate in pain control, chiefly in the late phase of neuropathic pain (30 days after BPA), probably by activating receptors located at the spinal and supra-spinal levels. This proportion is based on the fact that both systemic and intraplantar administration of the CB_1_ selective agonist displayed only a moderate effect on mechanical allodynia induced by BPA, when compared to the i.t. or i.c.v. routes. The significant inhibition of neuropathic pain by cannabinoid agonists when dosed via central routes could be related to increased cannabinoid receptor expression in the areas of the CNS involved in pain control [7], [8]. To explore the relevance of cannabinoid receptors in the central regulation of mechanical allodynia induced by BPA, we used the distinct approaches of blocking or knocking down CB_1_ and CB_2_ receptors in the spinal pathway. The results obtained by employing AS-ODN technology or the use of selective cannabinoid receptor antagonists confirmed the results obtained by the treatment of mice with cannabinoid selective agonists, supporting the major participation of CB_2_ receptors in the early phase, and both CB_1_ or CB_2_ receptors in the late phase after BPA surgery. The attenuation of the antinociceptive action of WIN 55,212-2 after antisense oligonucleotide or antagonist administration was reflected by the reduction in both CB_1_ and CB_2_ receptors in the spinal cord. Others studies have confirmed the modulation of nociceptive thresholds by cannabinoid receptors by treatment with selective antisense and antagonists, or by using CB_1_^−/−^ or CB_2_^−/−^ knockout mice [28], [29]. These studies, as well as a number of further investigations, have demonstrated the potential of antisense strategies in pain research [for reviews], [ see 30,31]. In general, antisense oligonucleotides use Watson–Crick base pairing to specifically hybridize to mRNA sequences complementary to those contained in the antisense molecule. For this hybridization to occur, the targeted mRNA sequence must be accessible to the oligonucleotide, and several other events must occur [32]. The mechanism by which oligonucleotides enter cells remains controversial, but probably involves fluid phase pinocytosis, receptor-mediated endocytosis, or both. Oligonucleotides must arrive at the mRNA intact, avoiding destruction in lysosomes and degradation by the various endo- and exonucleases present intracellularly and within serum and tissues [33]. Finally, the oligonucleotides must hybridize to target mRNAs with sufficient affinity and specificity, and evoke a mechanism of action leading to mRNA inactivation or destruction. Antisense oligonucleotides inhibit protein expression, in most cases, through RNase-H-mediated cleavage of target mRNA. RNase H is an endonuclease that recognizes RNA–DNA duplexes and selectively cleaves the RNA strand [34], [35]. The mechanism is catalytic: once an RNA molecule is cleaved, the antisense oligonucleotide is thought to dissociate from the duplex and become available to bind a second target mRNA molecule. In the present manuscript, the discrepancies observed between the treatments with antagonists and antisense oligonucleotides (i.t.) may be justified initially by a higher affinity and specificity of the antisense oligonucleotides in inhibiting spinal cannabinoid receptor expression, mainly in DRG neurons, as discussed earlier. Moreover, unlike antagonists that inhibit preformed cannabinoid receptors, treatment with antisense oligonucleotides shows a greater selectivity and efficacy in inhibiting protein translation of these receptors, which could justify, for instance, a greater effect of antisense treatment on reversing the analgesic effect of WIN 55,212-2.

The data presented herein show, for the first time that the brachial plexus avulsion model produced an increase in CB_1_ and CB_2_ mRNA expression levels at different time-points after surgery. Moreover, the mechanical allodynia produced by BPA was accompanied by an increase in glial cell activation and expression of p38 and JNK MAP kinases, associated with activation of the transcription factors CREB and p65 NF-κB. We reported that cannabinoid agonists display marked antinociceptive effects in the BPA mouse model, and that such actions are dependent on their ability to activate mainly CB_2_ receptors in the initial phase, and both CB_1_ and CB_2_ receptors in the late phase after BPA surgery. The cannabinoid agonist analgesic properties observed in the BPA model are related to the inhibition of glial cells and MAP kinase activation, associated with an enhancement of CREB and p65 NF-κB activation. Altogether, our findings suggest that cannabinoid agonists, acting via the activation of both CB_1_ and CB_2_ receptors, could represent attractive and useful strategies to control long-lasting neuropathic pain states, such as that observed after BPA. Furthermore, we believe that our study contributes to comprehending the role played by cannabinoid receptors following neuropathic injury.

## Materials and Methods

### Animals

Experiments were performed using female CD1 mice weighing 20 to 30 g (n = 4–6 per group). Animals were housed under conditions of optimum light, temperature and humidity (12 h light/dark cycle, 22±1°C, under 60–80% humidity), with food and water provided *ad libitum*. The studies reported in this manuscript followed the “Guide for the Care and Use of Laboratory Animals” as adopted and promulgated by the U.S. National Institutes of Health, and the ethical guidelines for the investigation of experimental pain in conscious animals [36]. The Ethics Committee of the Universidade Federal de Santa Catarina approved all the experimental procedures (046/CEUA/PRPE/2008). The number of animals and the intensity of noxious stimuli used were the minimum necessary to demonstrate the consistent effects of drug treatment.

### Surgical procedures for BPA

BPA was performed according to the methodology previously described and validated by Quintão et al. [37]. During surgery, the animals were anesthetized by inhalation with 3% isoflurane plus 3% oxygen. The right brachial plexus was approached through a longitudinal incision parallel to the clavicle, running from the sternum to the axillary region (1 cm). The subclavian vessels were located, and the lower trunk was dissected. The lower trunks were extorted by traction using forceps. In the sham-operated group, the brachial plexus was exposed and dissected without any lesion to the nerve. The tissue layers were then brought together, and the skin was closed with 4.0 silk suture strings (Ethicon, Edinburgh, UK).

### Hindpaw withdrawal response induced by von Frey hairs

To assess mechanical allodynia, mice were individually placed in clear Plexiglas boxes (9×7×11 cm) on elevated wire-mesh platforms to allow access to the ventral surface of the right hindpaw. The animals were acclimatized for 30 min before behavioral testing. The withdrawal response frequency was measured after 10 applications (duration of 1 s each) of von Frey hair 0.4 g (VFH; Stoelting, Chicago, IL). The 0.4 g VFH produces a mean withdrawal frequency of about 20%, which is considered to be an adequate value for the measurement of the mechanical withdrawal threshold in naïve or sham-operated mice, providing an adequate window to observe changes in mechanical sensation in either inflammatory or neuropathic mouse models of nociception. This choice was made considering that after BPA, both sensory and motor fibers of the forepaws are partially disrupted and the animals do not completely react to mechanical stimulation. To determine the basal mechanical thresholds, all the experimental groups were submitted to a pre-surgical evaluation (basal assessment) and they were re-evaluated at time 0 (before drug treatment), on the 5^th^ and 30^th^ days after surgery and at several time-points after agonist treatment (at 1, 2, 4, 6 and 8 h after treatment).

### Effect of cannabinoid agonists on the allodynia induced by BPA

The effects of cannabinoids on neuropathic pain following BPA was initially evaluated by using the non-selective agonist of CB_1_ and CB_2_ receptors WIN 55,212-2 (R-[2,3-dihydro-5-methyl-3(4-morpholinylmethyl)pyrrolo[1,2,3,-de]-1,4-benzoxazin-6-yl]-1-naphthalenyl methanone mesylate), dosed at different time intervals and routes of administration. As a first experimental approach, operated mice were treated with WIN 55,212-2 on the following schedule of administration: 10 and 30 µg/site, intraplantar (i.pl) in the right hindpaw; 3 and 5 mg/kg, intraperitoneally (i.p.); 1 and 5 µg/site, intrathecally (i.t.); or 1 and 5 µg/site, intracerebroventricularly (i.c.v.). Treatment with the agonists was performed at 5 or 30 days after surgery, and mechanical allodynia was evaluated as previously described. These time points were chosen on the basis of previous literature showing that these are suitable intervals of time to evaluate the acute (5^th^ day) and long-lasting (30^th^ day) changes evoked by BPA, without any interference of the surgical procedures [37], [38].

In another set of experiments, the operated mice received the CB_1_ selective agonist ACEA ([N-(2-chloroethyl)5,8,11,14-eicosaetraenamide]) (>1400-fold CB_1_ selectivity over CB_2_) (10 and 30 µg/site, i.pl; 1 and 10 mg/kg, i.p.; 10 and 30 µg/site, i.t.; or 10 and 30 µg/site, i.c.v.), or the CB_2_ selective agonist JWH-015 (2-methyl-1-propyl-1H-indol-3-yl)-1-naphthalenylmethanone) (>23-fold CB_2_ selectivity over CB_1_) (1 and 10 µg/site, i.pl; 1 and 10 mg/kg, i.p.; 1 and 10 µg/site, i.t.; or 1 and 10 µg/site, i.c.v.), administered on the 5^th^ or the 30^th^ day after surgery. The doses of agonists used in our study were selected on the basis of literature data or pilot experiments [10], [39]–[43].

### Influence of CB_1_ and CB_2_ selective antagonists on the analgesic effects elicited by a cannabinoid agonist

To confirm the effect of cannabinoid agonists in reducing the mechanical allodynia induced by BPA, different groups of mice were pre-treated with the selective CB_1_ AM 251 (3 mg/kg, i.p.) or CB_2_ AM 630 (1 mg/kg, i.p.) receptor antagonists, dosed 30 min prior to treatment with the non-selective cannabinoid agonist, WIN 55, 212-2 (3 mg/kg, i.p.), on the 5^th^ or 30^th^ day after surgery. Mechanical allodynia was measured with 0.4 g von Frey filament (VFH), as described above, at different time-points. The doses of antagonists used in our study were selected on the basis of literature data [3].

### Influence of CB_1_ and CB_2_ knockdown on the analgesic effects elicited by a cannabinoid agonist

To further confirm the effects of cannabinoids in neuropathic pain following BPA, different groups of mice received on the 2^nd^ or the 27^th^ day after BPA surgery, over a period of three days at 12 h intervals, i.t. injections of antisense oligodeoxynucleotides (AS-ODN) directed against the specific coding region of the CB_1_ (CB_1_ AS-ODN, sequence 5′-GCCGTCTAAGATCGACTT-3′) or CB_2_ receptor (CB_2_ AS-ODN, sequence 5′-CTGCTGAGCGCCCTGGAGAAC-3′). The control groups received i.t. injections of mismatch ODN control (MM AS-ODN, sequence 5′-GCCTGCTAGAATCGCATT-3′). AS-ODN (12.5 µg) was injected by the i.t. route in a volume of 5 µl (2.5 µg/µl). At the end of the third injection day, mice also received WIN 55,212-2 (3 mg/kg, i.p.) or vehicle (10 ml/kg, i.p.), 30 min after the last i.t. injection. Mechanical allodynia was measured with 0.4 g von Frey filament (VFH), as described above, at different time-points. To prove the efficacy of treatment with AS-ODN directed to CB_1_ and CB_2_ receptors, the cervical-thoracic portion (C4-T2) of the spinal cord of AS-ODN-treated mice was analysed immunohistochemically as described below.

### Effect of cannabinoid agonists on the activation of glial cells, MAP kinases and transcription factors pathways activated after BPA

To investigate the involvement of cannabinoids on the activation of glial cells, MAP kinases and transcription factors involved in neuropathic pain, we performed two treatment protocols. First, mice received a single i.p. injection of WIN 55,212-2 (3 mg/kg) on the 5^th^ and 30^th^ days after surgery. In the second protocol, mice received five treatments of WIN 55,212-2 (3 mg/kg, i.p.), starting on the 3^th^ or 27^th^ day after surgery (12/12 h), and 2 h after the last treatment (on the 5^th^ or 30^th^ day after surgery), mice were anesthetized with 7% chloral hydrate (10 ml/kg, i.p.) and perfused with PBS solution containing 4% paraformaldehyde (PFA). The cervical-thoracic portion of the spinal cord (C4 to T2) was collected and fixed in 4% paraformaldehyde in 0.2 M sodium phosphate, pH 7.4, for subsequent immunohistochemical analysis. The results obtained for treated operated mice were compared with those obtained for operated mice that had received vehicle only.

### Immunohistochemical studies

On the 5^th^ or the 30^th^ day following BPA, mice were anesthetized with 7% chloral hydrate (10 ml/kg, i.p.) and perfused with fresh 4% paraformaldehyde in 0.2 M sodium phosphate, pH 7.4. Immunohistochemical analysis were performed on paraplast®-embedded tissue sections (5 µm) of DRGs (C4-T2 spinal cord segment), cervical-thoracic spinal cord (C4-T2 spinal cord segment) and somatosensory cortex (cingulate cortex) using polyclonal rabbit anti-CB_1_ (1∶500), polyclonal rabbit anti-CB2 (1∶150), polyclonal goat anti-Iba-1 (ionized calcium binding adaptor molecule 1) (1∶200), monoclonal mouse anti-GFAP (glial fibrillary acidic protein) (1∶600), polyclonal rabbit anti-phospho-CREB (1∶200), polyclonal rabbit anti-phospho-p65 (NF-κB) (1∶100), monoclonal mouse anti-phospho-JNK (1∶200) and polyclonal rabbit anti-phospho-p38 (1∶60) antibodies. After quenching of endogenous peroxidase with 1.5% hydrogen peroxide in methanol (v/v) for 20 min, high-temperature antigen retrieval was performed by immersion of the slides in a water bath at 95 to 98°C in 10 mmol/L trisodium citrate buffer, pH 6.0, for 45 min. Subsequently, the sections were incubated overnight at 4°C with the primary antibodies. After incubation with the appropriate biotinylated secondary antibody, immune complexes were visualized with 0.05% 3,3′-diaminobenzidine tetrahydrochloride (DAB: Dako Cytomation, Glostrup, Denmark)+0.03% H_2_O_2_ in PBS (for the exact amount of time of 10 seconds;, the reaction was stopped by thorough washing in water and sections were counterstained with Harris' hematoxylin. Control and experimental tissues were placed on the same slide and processed under the same conditions. Besides staining untreated animals as negative controls, sections were incubated with isotype-matched primary antibodies of irrelevant specificity, or the primary antibody was omitted. Despite antigen retrieval, these controls resulted in little or no staining, principally due to the fact that peroxide pre-treatment (inactivation of endogenous peroxidase) appears to destroy the epitopes to which secondary antibody otherwise binds. Images were acquired using a Sight DS-5M-L1 digital camera connected to an Eclipse 50i light microscope (both from Nikon, Melville, NY, USA). Settings for image acquisition were identical for control and experimental tissues. Four microscopic fields (400×) per section of the DRG and of the dorsal horn of the spinal cord regions associated primarily with the termination of nociceptive primary afferents were captured and a threshold optical density that best discriminated staining from the background was obtained using ImageJ 1.36b imaging software (NIH, Bethesda, MD, USA). The nucleus proprius (lamina IV and V) and the ventral horn of the spinal cord were not evaluated. For all antibodies, analysis of the dorsal root of the spinal cord and the DRG, was performed using total pixel intensity, and data were expressed as arbitrary units. The cervical-thoracic portion of the spinal cord was used for expression studies, considering that it is the area responsible for the innervation of the brachial plexus.

### Determination of cannabinoid receptor expression by real-time quantitative-PCR

The expression of CB_1_R and CB_2_R mRNA was measured using quantitative RT-PCR assays. Separate groups of operated and sham-operated mice were killed on the 5^th^ or the 30^th^ day following BPA, and the ipsilateral dorsal root ganglion (DRG) and the spinal cord from the cervical portion (C4-T2 spinal cord segment) were isolated, dissected and frozen in liquid nitrogen and stored at −80°C. Total RNA from tissues was collected and extracted using the TRizol protocol and its concentration was determined using a NanoDrop 1100 (NanoDrop Technologies, Wilmington, DE, USA). The reverse transcription assay was carried out as described in the M-MLV Reverse Transcriptase protocol according to the manufacturer's instructions. cDNA (300 ng) was amplified in triplicate using TaqMan® Universal PCR Master Mix Kit with specific TaqMan Gene Expression target genes, with 3′ quencher MGB and FAM-labeled probes directed against the cDNA for the CB_1_ (Mm01212171_s1) and CB_2_ (Mm00438286_m1) receptors, as well as GAPDH (Mm03302249_g1), which was used as an endogenous control for normalization. The PCR reactions were performed in a 96-well Optical Reaction Plate (Applied Biosystems, Foster City, CA, USA). The thermocycler parameters were as follows: 50°C for 2 min, 95°C for 10 min, 50 cycles of 95°C for 15 sec, and 60°C for 1 min. Fluorescence was measured for each amplification cycle and the data were analyzed using the 2^−ΔΔCT^ method for relative quantification of expression. Expression of the target genes was calibrated against conditions found in control animals, i.e., those which received the vehicle.

### General drug administration procedures

The i.t. injections were performed in conscious animals to avoid possible anesthetic interference, according to the method described by Hylden and Wilcox [44], with some modifications. A needle connected to a microsyringe by polyethylene tubing was introduced through the skin, and a volume of 5 µl of vehicle (control) or one of the cannabinoid agonists was injected between the L5 and L6 vertebral spaces. For i.c.v. injections, the animals were slightly anesthetized by inhalation with 3% isoflurane plus 3% oxygen, and a free-hand injection of a 2 µl volume of agonists or vehicle was injected directly into the left ventricle of the brain, as described previously by Laursen and Belknap [45].

### Drugs and reagents

R-[2,3-dihydro-5-methyl-3(4-morpholinylmethyl)pyrrolo[1,2,3,-de]-1,4-benzoxazin-6-yl]-1-naphthalenyl methanone mesylate (WIN 55,212-2), (2-methyl-1-propyl-1H-indol-3-yl)-1-naphthalenylmethanone (JWH-015), [N-(2-chloroethyl)5,8,11,14-eicosaetraenamide] (ACEA), and paraplast® were purchased from Sigma Chemical Company (St. Louis, MO, USA). The 6-iodo-2-methyl-1-[2-(4-morpholinyl)ethyl]-1H-indol-3-y l](4-methoxyphenyl) methanone (AM 630) and N-(piperidin-1-yl)-5-(4-iodophenyl)-1-(2,4-dichlorophenyl)-4-methyl-1H-pyrazole-3-carboxamide (AM 251) were purchased from Tocris Bioscience (Ellisville, MO, USA). Isoflurane was obtained from Cristália (São Paulo, SP, Brazil). Chloral hydrate was purchased from Vetec (Rio de Janeiro, RJ, Brazil). The antisense oligodeoxynucleotides specific for CB_1_ (CB_1_ AS-ODN), CB_2_ (CB_2_ AS-ODN) or the mismatch (MM AS-ODN) were purchased from Prodimol Biotecnologia (Belo Horizonte, MG, Brazil). TRizol was purchased from Invitrogen (Carlsbad, CA, USA), Oligo dT, M-MLV reverse transcriptase, dNTP, DTT and Tris-HCl and RNAsin from Promega (Madison, WI, USA) PCR master mix kit and FAM-labeled probes for CB_1_, CB_2_ and GAPDH from Applied Biosystems (Foster City, CA, USA), phosphate buffered saline (PBS), trisodium citrate, hydrogen peroxide, Harris' hematoxylin, MgCl_2_, KCl, paraformaldehyde, ethanol, xylene, methanol, acetone, dimethylsulfoxide and Tween 80 from Merck & Co., Inc. (Whitehouse Station, NJ, USA). Polyclonal rabbit anti-CB_1_ and polyclonal rabbit anti-CB_2_ antibodies were purchased from Cayman Chemicals (Ann Arbor, MI, USA), monoclonal mouse anti (phospho)-JNK from Santa-Cruz Biotechnology Inc. (Santa Cruz, CA, USA). Polyclonal rabbit anti-phosphorylated p38 (Thr180 and Tyr182), monoclonal mouse anti-GFAP, polyclonal rabbit anti-p-CREB (Ser133) and polyclonal rabbit anti-p-p65 NF-κB (Ser276) were obtained from Cell Signaling Technology (Beverly, MA, USA). Polyclonal goat anti-Iba-1 was purchased from Abcam (Cambridge, MA, USA). Streptavidin-horseradish peroxidase and 3,3′-diaminobenzidine (DAB) were purchased from Dako (Glostrup, Denmark). WIN 55,212-2 was dissolved in 2% DMSO and 2% Tween 80. Stock solutions were prepared with 100% ethanol for JWH-015 and 100% DMSO for ACEA. The final concentration of ethanol or DMSO never exceeded 5%, which had no effect *per se* in our protocols. The control groups received the respective vehicle used to dissolve the drugs.

### Statistical analysis

The results are presented as the mean ± SEM of four to six animals for each experimental group. For the behavioral nociception assays, the percentages of inhibition are reported as the difference (in percentage) between the areas under the time-response curve of the test group in relation to the corresponding control group. Statistical comparison of data was performed by two-way ANOVA for repeated measures followed by Bonferroni's post-hoc test (treatment and time variable) or one-way ANOVA followed by Bonferroni's post-hoc test, depending on the experimental protocol. P values less than 0.05 (p<0.05) were considered significant. Statistical analyses were performed using GraphPad Prism version 5.00 software (GraphPad Software, San Diego, CA, USA).

## Supporting Information

Figure S1 **The CB_1_ receptor (CB_1_) and CB_2_ receptor (CB_2_) immunoreactivity in CNS structures of mice submitted to brachial plexus avulsion (BPA).** CB_1_ and CB_2_ receptor expression were evaluated in the DRG, the dorsal horn of the spinal cord and the cingulate cortex in N (naïve mice), Sh 5 d (sham-operated group 5 days after BPA), Op 5 d (operated group 5 days after BPA), Sh 30 d (sham-operated group 30 days after BPA) and Op 30 d (operated group 30 days after BPA). The scale bar corresponds to 50 µm and applies throughout.
(TIF)

Figure S2 **Immunoreactivity against glial cells, MAP kinases and transcription factors in the dorsal horn of the spinal cord of mice submitted to brachial plexus avulsion (BPA).** Immunostaining of activated microglia (Iba-1), astrocytes (GFAP), phospho-p38 (p-p38) and phospho-JNK (p-JNK) MAP kinases, phospho-CREB (p-CREB) and phospho-p65 NF-κB (p-p65) transcription factors in N (naïve mice), Sh 5 d (sham-operated group 5 days after BPA), Op 5 d (operated group 5 days after BPA), Sh 30 d (sham-operated group 30 days after BPA) and Op 30 d (operated group 30 days after BPA). The scale bar corresponds to 50 µm and applies throughout.
(TIF)

Figure S3 **Local administration of cannabinoid agonists inhibits mechanical allodynia induced by brachial plexus avulsion (BPA) in mice.** Response frequency of the right hindpaw assessed at several time-points in operated (BPA) mice treated with the non-selective CB_1_ and CB_2_ agonist, WIN 55,212-2 (10–30 µg/paw, i.pl.), the selective CB_1_ agonist, ACEA (10–30 µg/paw, i.pl.), the selective CB_2_ agonist, JWH-015 (1–10 µg/paw, i.pl.) or vehicle (20 µl/paw, i.pl.), administered on the 5^th^ (A, B and C) and 30^th^ (D, E and F) day after surgery. Data are expressed as mean ± SEM (n = 4–6/group). *p<0.05, significantly different from the operated group treated with vehicle (two-way ANOVA with Bonferroni's *post hoc* test). B, Baseline withdrawal threshold before surgery. 0, baseline withdrawal threshold after surgery on the day of the experiment (5^th^ and 30^th^ days).
(TIF)

Figure S4 **Effect of treatment with selective antisense oligodeoxinucleotide (AS-ODN) on CB_1_ and CB_2_ receptor expression in the spinal cord of mice.** Immunostaining of activated of CB_1_ and CB_2_ receptors in operated (Op) mice received an i.t. injection twice daily of mismatch (MM) AS-ODN (12.5 µg/site), CB_1_ AS-ODN (12.5 µg/site) or CB_2_ AS-ODN (12.5 µg/site) on the 5^th^ and 30^th^ day after brachial plexus avulsion (BPA). The scale bar corresponds to 50 µm and applies throughout.
(TIF)

Figure S5 **Effect of cannabinoid agonist on the expression of glial cells, MAP kinases and transcription factors in the dorsal horn of the spinal cord after brachial plexus avulsion (BPA) in mice.** Immunostaining of activated microglia (Iba-1), astrocytes (GFAP), phospho-p38 (p-p38) and phospho-JNK (p-JNK) MAP kinases, phospho-CREB (p-CREB) and phospho-p65 NF-κB (p-p65) transcription factors in operated mice treated with vehicle (10 ml/kg, i.p.), acute (once) or long-term (five times) treatment with WIN 55,212-2 (3 mg/kg, i.p.) on the 5^th^ and 30^th^ days after BPA. The scale bar corresponds to 50 µm and applies throughout. JNK: c-Jun N-terminal kinase; CREB: cAMP response element-binding protein; p65 NF-κB: nuclear phospho-p65 nuclear factor-κB (NF-κB).
(TIF)

Table S1 **Behavioral effects of cannabinoids agonists in the mouse tetrad assay.**
(DOC)

Supplementary Methods **Tetrad behavioral assessment.**
(DOC)
