# Supplementary figures and images for: Cannabinoid Agonists Inhibit Neuropathic Pain Induced by Brachial Plexus Avulsion in Mice by Affecting Glial Cells and MAP Kinases
Source: PLoS One. 2011 Sep 13;6(9):e24034. doi: 10.1371/journal.pone.0024034 (PMC3172222; doi:10.1371/journal.pone.0024034)

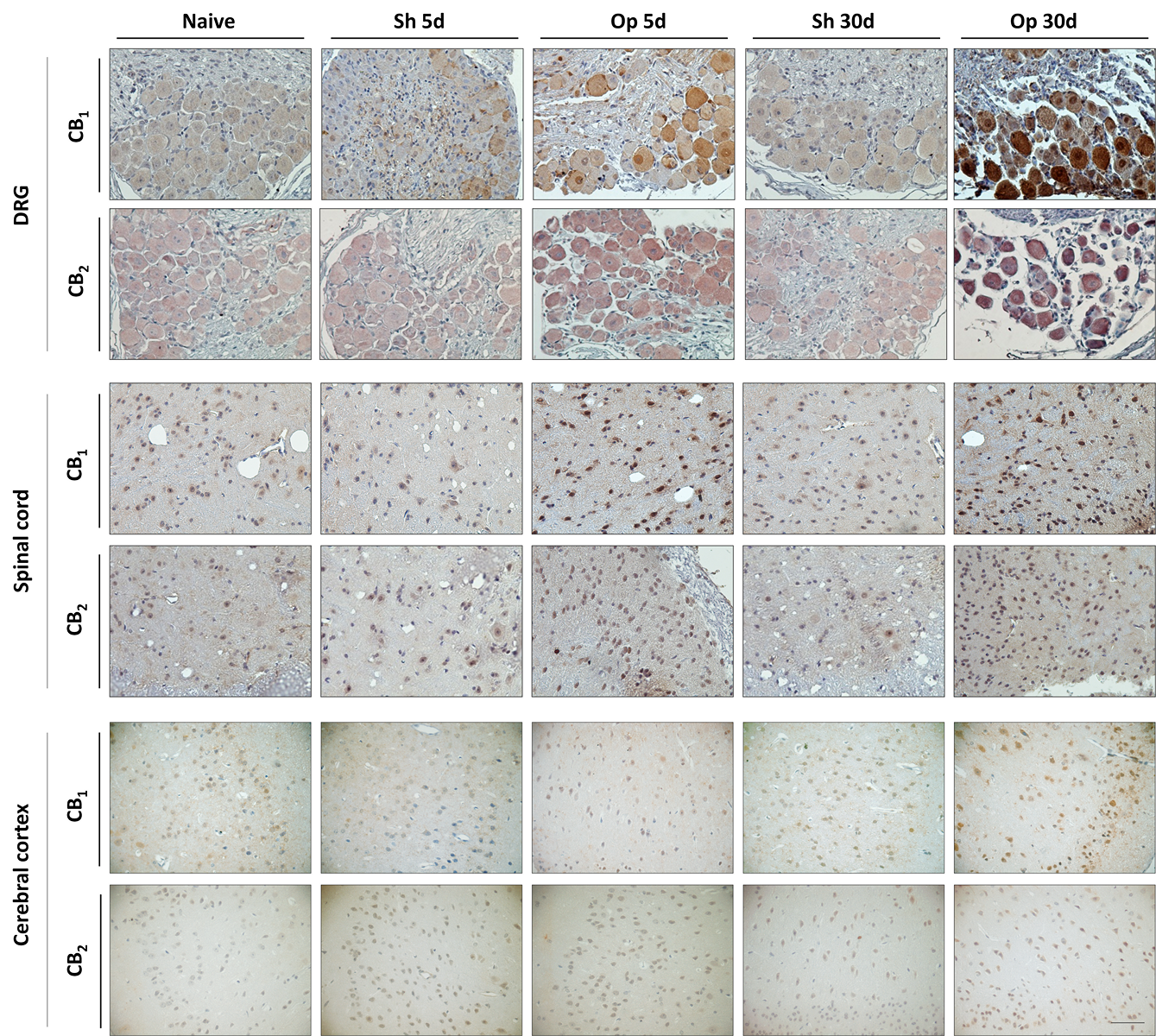

Supplement: Figure S1 — The CB1 receptor (CB1) and CB2 receptor (CB2) immunoreactivity in CNS structures of mice submitted to brachial plexus avulsion (BPA). CB1 and CB2 receptor expression were evaluated in the DRG, the dorsal horn of the spinal cord and the cingulate cortex in N (naïve mice), Sh 5 d (sham-operated group 5 days after BPA), Op 5 d (operated group 5 days after BPA), Sh 30 d (sham-operated group 30 days after BPA) and Op 30 d (operated group 30 days after BPA). The scale bar corresponds to 50 µm and applies throughout. (TIF) [file pone.0024034.s001.tif]

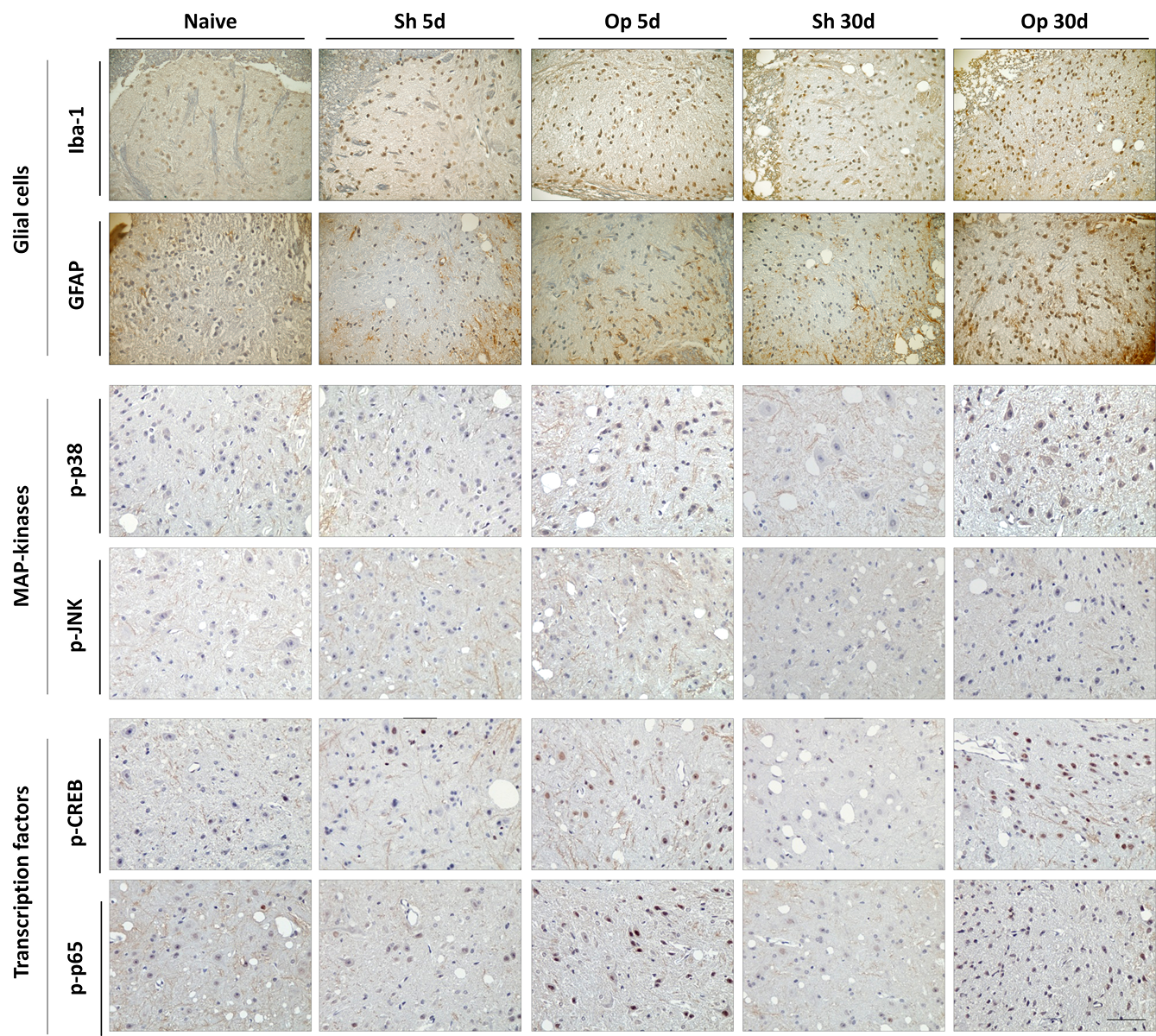

Supplement: Figure S2 — Immunoreactivity against glial cells, MAP kinases and transcription factors in the dorsal horn of the spinal cord of mice submitted to brachial plexus avulsion (BPA). Immunostaining of activated microglia (Iba-1), astrocytes (GFAP), phospho-p38 (p-p38) and phospho-JNK (p-JNK) MAP kinases, phospho-CREB (p-CREB) and phospho-p65 NF-κB (p-p65) transcription factors in N (naïve mice), Sh 5 d (sham-operated group 5 days after BPA), Op 5 d (operated group 5 days after BPA), Sh 30 d (sham-operated group 30 days after BPA) and Op 30 d (operated group 30 days after BPA). The scale bar corresponds to 50 µm and applies throughout. (TIF) [file pone.0024034.s002.tif]

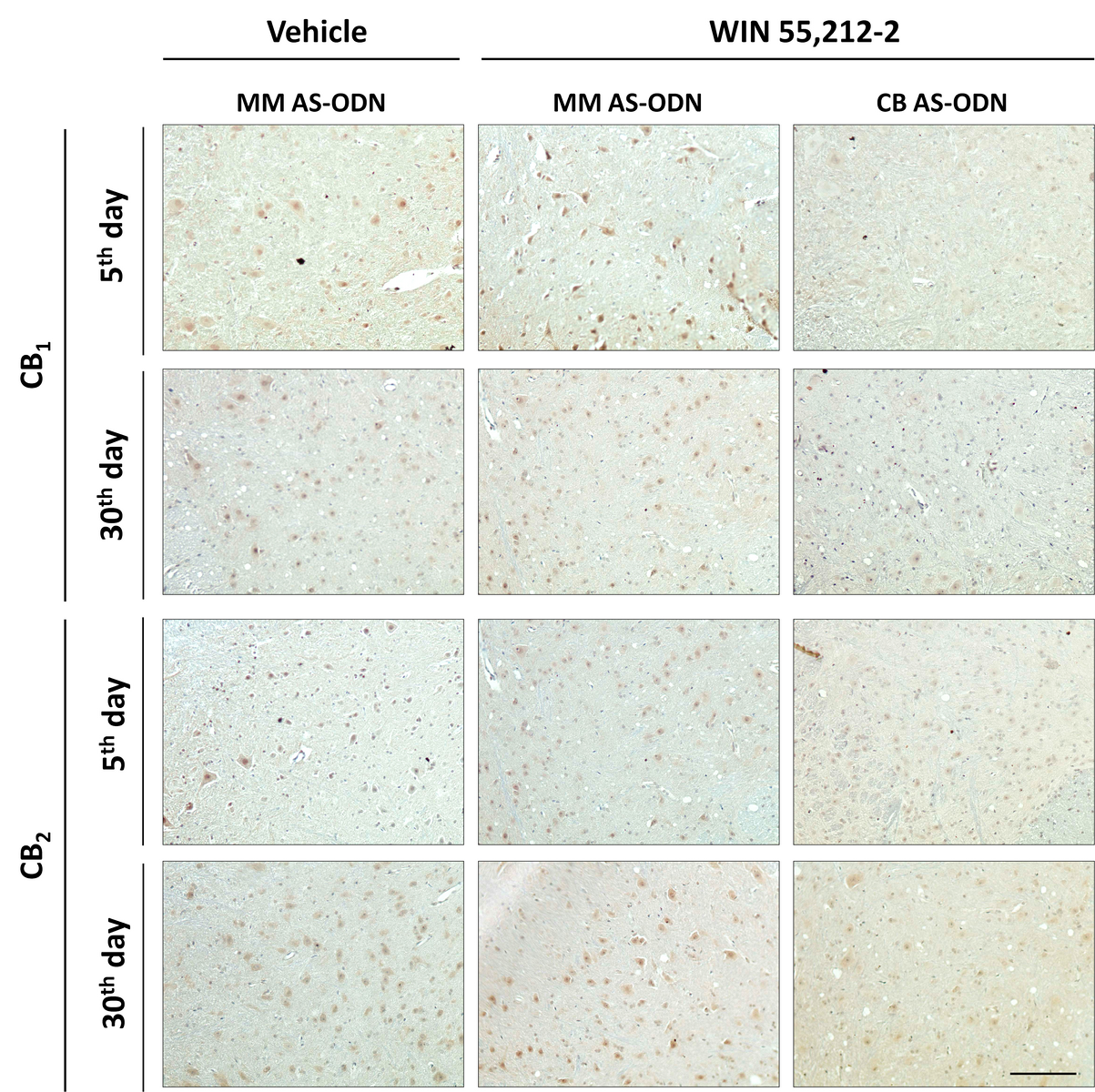

Supplement: Figure S4 — Effect of treatment with selective antisense oligodeoxinucleotide (AS-ODN) on CB1 and CB2 receptor expression in the spinal cord of mice. Immunostaining of activated of CB1 and CB2 receptors in operated (Op) mice received an i.t. injection twice daily of mismatch (MM) AS-ODN (12.5 µg/site), CB1 AS-ODN (12.5 µg/site) or CB2 AS-ODN (12.5 µg/site) on the 5th and 30th day after brachial plexus avulsion (BPA). The scale bar corresponds to 50 µm and applies throughout. (TIF) [file pone.0024034.s004.tif]

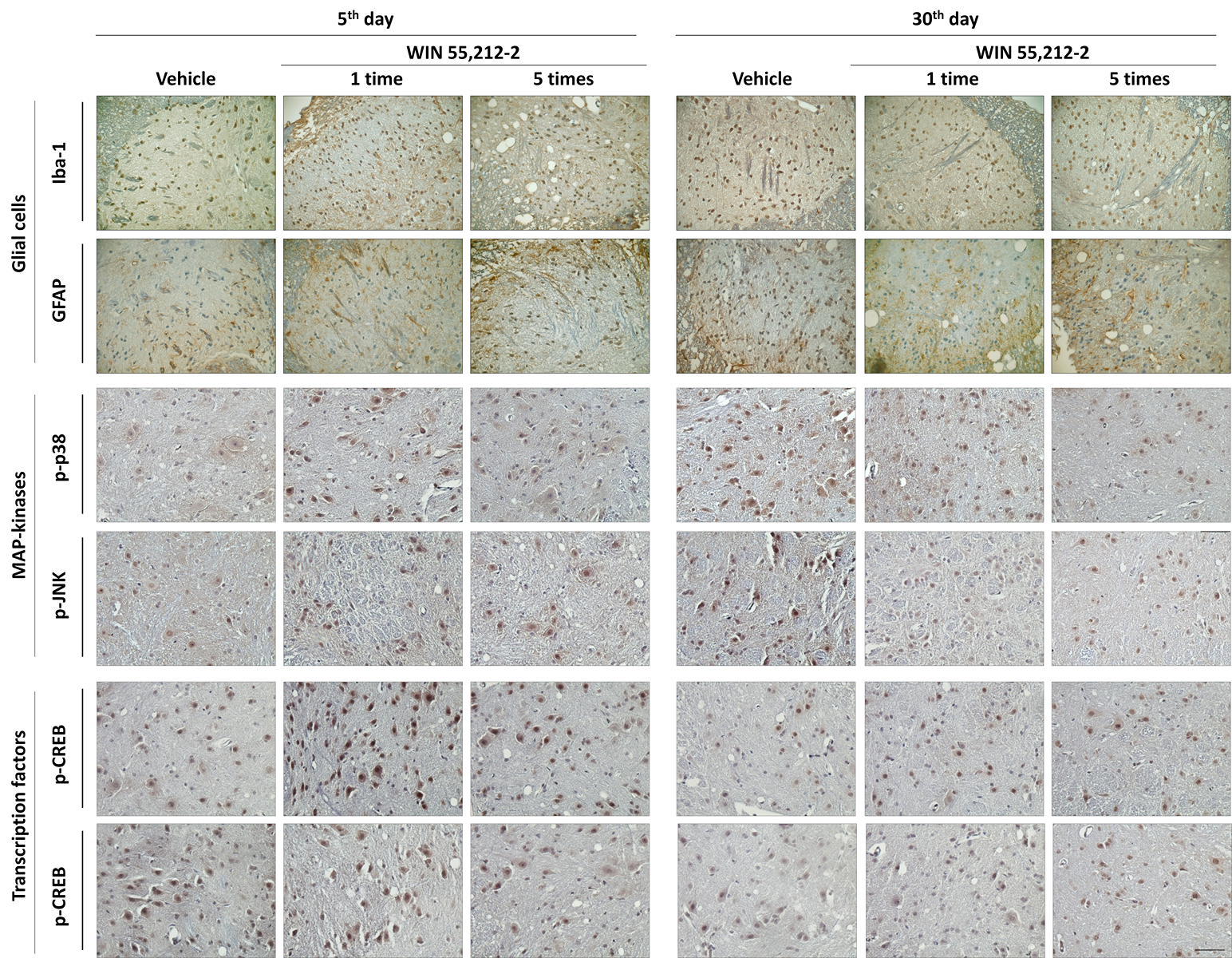

Supplement: Figure S5 — Effect of cannabinoid agonist on the expression of glial cells, MAP kinases and transcription factors in the dorsal horn of the spinal cord after brachial plexus avulsion (BPA) in mice. Immunostaining of activated microglia (Iba-1), astrocytes (GFAP), phospho-p38 (p-p38) and phospho-JNK (p-JNK) MAP kinases, phospho-CREB (p-CREB) and phospho-p65 NF-κB (p-p65) transcription factors in operated mice treated with vehicle (10 ml/kg, i.p.), acute (once) or long-term (five times) treatment with WIN 55,212-2 (3 mg/kg, i.p.) on the 5th and 30th days after BPA. The scale bar corresponds to 50 µm and applies throughout. JNK: c-Jun N-terminal kinase; CREB: cAMP response element-binding protein; p65 NF-κB: nuclear phospho-p65 nuclear factor-κB (NF-κB). (TIF) [file pone.0024034.s005.tif]
