# Supplementary material for: Cannabinoid Agonists Inhibit Neuropathic Pain Induced by Brachial Plexus Avulsion in Mice by Affecting Glial Cells and MAP Kinases
Source: PLoS One. 2011 Sep 13;6(9):e24034. doi: 10.1371/journal.pone.0024034 (PMC3172222; doi:10.1371/journal.pone.0024034)
Supplement: Table S1 — Behavioral effects of cannabinoids agonists in the mouse tetrad assay. (DOC) [file pone.0024034.s006.doc]

**Supplementary Table 1.** Behavioral effects of cannabinoids agonists in the mouse tetrad assay.

| **Drug** | **Dose (mg/kg)** | **Time (min)** | **Locomotor activity (s)** | **Catalepsy (s)** | **Rectal temperature (°C)** | **Tail flick latency (s)** |
| --- | --- | --- | --- | --- | --- | --- |
| **Vehicle** | - | 30 | 118. 2 ± 1.35 | 4.25 ± 0.85 | 37.48 ± 0.15 | 13.38 ± 1.91 |
|  |  | 60 | 118.6 ± 1.16 | 3.45 ± 2.13 | 37.24 ± 0.30 | 12.89 ± 1.15 |
|  |  | 120 | 120.0 ± 0.0 | 3.40 ± 1.07 | 37.52 ± 0.26 | 11.57 ± 1.65 |
| **WIN 55,212-2** | 5 | 30 | 115.8 ± 3.2 | 12.75 ± 4.32 | 35.8 ± 0.31 | 10.35 ± 0.46 |
|  |  | 60 | 117.4 ± 1.9 | 8.25 ± 5.46 | 36.3 ± 0.20 | 10.86 ± 0.70 |
|  |  | 120 | 119.0 ± 1.0 | 10.75 ± 1.70 | 37.44 ± 0.28 | 9.92 ± 0.90 |
| **ACEA** | 10 | 30 | 109.4 ± 10.6 | 2.80 ± 1.31 | 37.6 ± 0.39 | 10.36 ± 1.39 |
|  |  | 60 | 120.0 ± 0,0 | 3.60 ± 1.28 | 37.6 ± 0.26 | 9.66 ± 2.67 |
|  |  | 120 | 120.0 ± 0.0 | 5.80 ± 1.24 | 37.6 ± 0.25 | 8.14 ± 1.24 |
| **JWH-015** | 10 | 30 | 104.0 ± 13.6 | 4.20 ± 1.01 | 37.8 ± 0.13 | 11,6 ± 0.90 |
|  |  | 60 | 107.2 ± 8.33 | 3.80 ± 0.96 | 37.4 ± 0.35 | 10.78 ± 1.15 |
|  |  | 120 | 106.4 ± 13.6 | 3.80 ± 1.90 | 37.5 ± 0.22 | 12.0 ± 1.67 |

Values represent the mean ± SEM of 4 to 6 animals per group.
