# Supplementary material for: Cannabinoid Agonists Inhibit Neuropathic Pain Induced by Brachial Plexus Avulsion in Mice by Affecting Glial Cells and MAP Kinases
Source: PLoS One. 2011 Sep 13;6(9):e24034. doi: 10.1371/journal.pone.0024034 (PMC3172222; doi:10.1371/journal.pone.0024034)
Supplement: Supplementary Methods — Tetrad behavioral assessment. (DOC) [file pone.0024034.s007.doc]

**Supplementary Methods**

**Tetrad behavioral assessment**

*Hypothermia:* Core temperature in the mice were measured using a digital clinic thermometer (BD Basics, USA), which was lubricated and inserted into the rectum to a constant depth of 2 cm. Data were recorded before, 30, 60 and 120 min after systemic treatment with WIN 55,212-2 (5 mg/kg, i.p.), JWH-015 (10 mg/kg, i.p.) or ACEA (10 mg/kg, i.p.), and the results were compared to those from vehicle (10 ml/kg, i.p.) treated mice.

*Catalepsy:* Mice were hung by their front paws over a 0.5 cm diameter horizontal glass bar, supported 4 cm above the floor by two 8 x 8 cm pieces of metal, and the time taken for the mouse to move off the ring was measured with a cut-off of 60 s. Data are expressed as the total time spent on the ring during which the animal remained motionless. Latencies were measured before, 30, 60 and 120 min after systemic treatment with WIN 55,212-2 (5 mg/kg, i.p.), JWH-015 (10 mg/kg, i.p.) and ACEA (10 mg/kg, i.p.), and the results were compared to those from vehicle (10 ml/kg, i.p.) treated mice.

*Locomotor activity:* Motor coordination was evaluated using the rota-rod test (Ugo Basile, Italy), in which animals were required to walk against the motion of a rotating drum, with a fixed rotational speed of 4 rpm. Mice were placed in the recording chambers (40 (w) x 30 (d) x 38 (h) cm) and training sessions were carried out one and two days prior to experimentation until animals were able to remain on the rota rod for at least 120 s (chosen to evaluate locomotor activity). The evaluation was made before, 30, 60 and 120 min after systemic treatment with WIN 55,212-2 (5 mg/kg, i.p.), JWH-015 (10 mg/kg, i.p.) and ACEA (10 mg/kg, i.p.), and the results compared to those from vehicle (10 ml/kg, i.p.) treated mice.

*Analgesia:* To measure the response to acute noxious thermal stimuli, a radiant heat analgesiometer (Albarsch, Tail-Flick Analgesia Meter) was used to measure latencies for tail withdrawal. All the animals were submitted to the test for determining the basal thermal threshold, and were then reevaluated 30, 60 and 120 min after systemic treatment with WIN 55,212-2 (5 mg/kg, i.p.), JWH-015 (10 mg/kg, i.p.) or ACEA (10 mg/kg, i.p.), and the results compared to those from vehicle (10 ml/kg, i.p.) treated mice. A maximum cut-off of 20 s was used to prevent tissue damage and sensitization in subsequent readings.
